# Supplementary material for: Evaluating the impact of the Bolsa Familia conditional cash transfer program on premature cardiovascular and all-cause mortality using the 100 million Brazilian cohort: a natural experiment study protocol
Source: BMJ Open. 2020 Nov 20;10(11):e039658. doi: 10.1136/bmjopen-2020-039658 (PMC7682454; doi:10.1136/bmjopen-2020-039658)
Supplement: Author's manuscript [file bmjopen-2020-039658.draft_revisions.pdf]

BMJ Open is committed to open peer review. As part of this commitment we make the peer review history of every article we publish publicly available.

When an article is published we post the peer reviewers' comments and the authors' responses online. We also post the versions of the paper that were used during peer review. These are the versions that the peer review comments apply to.

The versions of the paper that follow are the versions that were submitted during the peer review process. They are not the versions of record or the final published versions. They should not be cited or distributed as the published version of this manuscript.

BMJ Open is an open access journal and the full, final, typeset and author-corrected version of record of the manuscript is available on our site with no access controls, subscription charges or pay-per-view fees (http://bmjopen.bmj.com).

If you have any questions on BMJ Open's open peer review process please email info.bmjopen@bmj.com

BMJ Open

Evaluating the impact of the Bolsa Familia conditional cash transfer program on premature cardiovascular and all-cause mortality using the 100 million Brazilian cohort: A natural experiment study protocol

Journal:	*BMJ Open*
Manuscript ID	bmjopen-2020-039658
Article Type:	Protocol
Date Submitted by the Author:	22-Apr-2020

Complete List of Authors:	Pescarini, Julia; Fundação Oswaldo Cruz, Centro de Integração de Dados e Conhecimentos para Saúde (Cidacs)<br>Craig , Peter ; University of Glasgow, MRC/CSO Social & Public Health Sciences Unit<br>Allik, Mirjam; University of Glasgow, MRC/CSO Social & Public Health Sciences Unit<br>Amorim, Leila; Universidade Federal da Bahia, Institute of Mathematics and Statistics<br>Ali, Sanni; London School of Hygiene & Tropical Medicine<br>Smeeth, Liam; London School of Hygiene & Tropical Medicine, Epidemiology and Population Health<br>Barreto, Mauricio; Universidade Federal da Bahia, Instituto de Saúde Coletiva; Fundação Oswaldo Cruz, Centro de Integração de Dados e Conhecimentos para Saúde (Cidacs)<br>Leyland, Alastair; University of Glasgow, MRC/CSO Social & Public Health Sciences Unit<br>Aquino, Estela M. L.; Universidade Federal da Bahia, Instituto de Saúde Coletiva<br>Katikireddi, Srinivasa; University of Glasgow School of Life Sciences, MRC/CSO Social & Public Health Sciences Unit

Keywords:	Health policy < HEALTH SERVICES ADMINISTRATION & MANAGEMENT, PUBLIC HEALTH, Cardiac Epidemiology < CARDIOLOGY

SCHOLARONE™
Manuscripts

I, the Submitting Author has the right to grant and does grant on behalf of all authors of the Work (as defined in the below author licence), an exclusive licence and/or a non-exclusive licence for contributions from authors who are: i) UK Crown employees; ii) where BMJ has agreed a CC-BY licence shall apply, and/or iii) in accordance with the terms applicable for US Federal Government officers or employees acting as part of their official duties; on a worldwide, perpetual, irrevocable, royalty-free basis to BMJ Publishing Group Ltd ("BMJ") its licensees and where the relevant Journal is co-owned by BMJ to the co-owners of the Journal, to publish the Work in this journal and any other BMJ products and to exploit all rights, as set out in our licence.

The Submitting Author accepts and understands that any supply made under these terms is made by BMJ to the Submitting Author unless you are acting as an employee on behalf of your employer or a postgraduate student of an affiliated institution which is paying any applicable article publishing charge ("APC") for Open Access articles. Where the Submitting Author wishes to make the Work available on an Open Access basis (and intends to pay the relevant APC), the terms of reuse of such Open Access shall be governed by a Creative Commons licence – details of these licences and which Creative Commons licence will apply to this Work are set out in our licence referred to above.

Other than as permitted in any relevant BMJ Author's Self Archiving Policies, I confirm this Work has not been accepted for publication elsewhere, is not being considered for publication elsewhere and does not duplicate material already published. I confirm all authors consent to publication of this Work and authorise the granting of this licence.

For peer review only

Evaluating the impact of the Bolsa Familia conditional cash transfer program on premature cardiovascular and all-cause mortality using the 100 million Brazilian cohort: A natural experiment study protocol

Julia M Pescarini[1*], Peter Craig[2], Mirjam Allik[2], Leila Amorim[3], Sanni Ali[4], Liam Smeeth[4,5], Mauricio L Barreto[1], Alastair H Leyland[2], Estela ML Aquino[6**], Srinivasa Vittal Katikireddi[2**].

1. Centro de Integração de Dados e Conhecimentos para Saúde (Cidacs), Fundação Oswaldo Cruz, Salvador, Brazil.
2. MRC/CSO Social & Public Health Sciences Unit, University of Glasgow, Glasgow, United Kingdom.
3. Institute of Mathematics and Statistics, Universidade Federal da Bahia, Salvador, Brazil.
4. Department of Non-Communicable Disease Epidemiology, London School of Hygiene & Tropical Medicine, London, United Kingdom.
5. Health Data Research (HDR), London, United Kingdom.
6. Instituto de Saúde Coletiva, Universidade Federal da Bahia, Salvador, Brazil.

***Corresponding Author:** Julia M. Pescarini
E-mail: juliapescarini@gmail.com (JMP)
Postal address: Centro de Integração de Dados e Conhecimentos para Saúde (Cidacs), Fundação Oswaldo Cruz. Edf. Tecnocentro – R. Mundo, 121 – Trobogy, Salvador – Bahia/Brazil. Postal code: 41745-715.
Telephone: +71 982608344

****Co-senior authors**

Word Count: (3,592 words)

Keywords: Cardiovascular diseases, quasi-experiment, social protection, social policies, cash-transfers.

For peer review only - http://bmjopen.bmj.com/site/about/guidelines.xhtml

Abstract

Introduction

Brazil's Bolsa Familia Programme (BFP) is the world's largest conditional cash transfer scheme. We shall use a large cohort of applicants for different social programmes to evaluate the effect of BFP receipt on premature all-cause and cardiovascular mortality.

Methods and analysis

We will identify BFP-recipients and non-recipients among new applicants from 2004-2015 in the 100 Million Brazilian Cohort, a database of 114 million individuals containing socio-demographic and mortality information of applicants to any Brazilian social programme. For individuals applying from 2011, when we have better recorded income data, we shall compare premature (age 30-69) cardiovascular and all-cause mortality among BFP-recipients and non-recipients using regression discontinuity design (RDD) with household monthly per capita income as the forcing variable. Effects will be estimated using survival models accounting for individual follow-up. To test the sensitivity of our findings, we will estimate models with different bandwidths, include potential confounders as covariates in the survival models, and restrict our data to locations with the most reliable data. In addition, we will estimate the effect of BFP on studied outcomes using propensity score risk-set matching, separately for individuals that applied ≤2010 and >2011, allowing comparability with RDD. Analyses will be stratified by geographical region, gender, race/ethnicity and socioeconomic position. We will investigate differential impacts of BFP and the presence of effect modification for a combination of characteristics, including gender and race/ethnicity.

Ethics and dissemination

The study was approved by the ethics committees of Oswaldo Cruz Foundation and the University of Glasgow College of Medicine and Veterinary Life Sciences. The deidentified dataset will be provided to researchers, and data analysis will be performed in a safe computational environment without internet access. Study findings will be published in high quality peer-reviewed research articles. The published results will be disseminated in the social media and to policy makers.

For peer review only - http://bmjopen.bmj.com/site/about/guidelines.xhtml

Article Summary

Strengths and limitations of this study

- Few previous studies of conditional cash-transfer programs have investigated impacts on adult health, or on premature death by CVD, using individual level data on exposures and outcomes.
- We exploit a nationwide linkage of social policy and health datasets to evaluate the largest CCT in the world in one of the most unequal countries.
- We use natural experimental approaches to estimate the effect of BFP that control for both observed and unobserved differences between recipients and non-recipients.
- Limitations associated with the use of routinely collected data include under-ascertainment of deaths, imperfect measurement of incomes, and a restricted range of covariates.
- The period of follow-up is limited to ten years, so may be insufficient to observe long-term impacts, including life course effects of improving socio-economic conditions in childhood.

For peer review only

For peer review only - http://bmjopen.bmj.com/site/about/guidelines.xhtml

Introduction

Conditional cash transfer (CCT) programmes have been widely implemented since the 1990s in the Americas, aiming to reduce poverty among groups largely excluded from previous social policies[1]. A World Bank Study using data from 79 countries suggested that such programmes have reduced absolute poverty (people living with <= US$1.90 per capita per day) by 36% and relative poverty (the poorest 20% in each country) by 8% [2]. CCT programs impose additional requirements on recipients, beyond a test of income, most commonly a health and/or education component that targets children and pregnant/breastfeeding women [1]. Therefore, the evidence of the impact of cash transfer programmes on health derives largely (but not exclusively) from studies evaluating the impact on food availability, nutrition, child and maternal health [3-5].

In low- and middle-income countries, cardiovascular diseases (CVD) still lead as the number one cause of death among non-communicable diseases (NCD), accounting for 9.6 million deaths in 2017, but mortality rates have been mainly increasing in middle-income countries [6, 7]. A recent systematic review notes low socioeconomic status, high alcohol intake, obesity, diabetes, hypertension, physical inactivity and smoking are the main modifiable factors associated with early mortality due to CVD [8]. Premature mortality (i.e., death among persons 30 to 69 years of age) is a particularly important indicator for monitoring the implementation of effective public policies for disease prevention and control [9].

In Brazil, poverty is largely concentrated among women, Blacks, and individuals living in rural areas [10]. The epidemiological transition in the country occurred heterogeneously across regions, and the overall increase in life expectancy was accompanied by a greater decrease in CVD mortality in regions with better socioeconomic conditions.[11, 12] In 2013, a nationwide survey highlighted a higher prevalence of risk factors for CVD (i.e., smoking, heavy alcohol use, physical inactivity and sedentary lifestyle, as well as the lower consumption of fruits and vegetables) among Brazilians from Non-White ethnic backgrounds and those with lower education, illustrating the relationship between poverty and racial/ethnic, gender and income disparities in health [13].

The Bolsa Familia Program (BFP), the world's largest CCT, benefits over 13 million families a year and has helped to reduce poverty and inequality in Brazil [14-16]. Although the programme has had large effects on child and maternal mortality, its impact on NCDs, especially on cardiovascular deaths, remains unknown. Therefore, the aims of our study are to investigate the effects of the BFP on CVD (ischaemic heart disease and cerebrovascular disease) mortality and all-cause mortality, and to assess how these effects differ by socioeconomic position, race/ethnicity, urbanicity and region.

Our detailed objectives are:

1. To estimate the causal effect of BFP on premature cardiovascular disease mortality

For peer review only - http://bmjopen.bmj.com/site/about/guidelines.xhtml

2. To estimate the causal effect of BFP on premature ischaemic heart disease and cerebrovascular disease mortality

3. To estimate the causal effect of BFP on all-cause premature mortality.

4. To investigate whether the causal effects of BFP on cardiovascular and all-cause mortality differ by population subgroups, including gender, educational attainment, race/ethnicity, geographical region, urbanicity, and socioeconomic position.

5. To explore how combinations of selected social characteristics influence the causal effects of BFP on the above outcomes, adopting an intersectionality perspective.

Methods and analysis

Study design

This study will be analysed as a retrospective, dynamic and open cohort, linking data from the baseline registries of individuals in the 100 Million Brazilian Cohort, with data on Bolsa Familia Program intervention receipt and individual level mortality records for the whole country.

Intervention

We report key implementation characteristics as per the TIDieR-PHP template [17]. BFP was implemented in 2004 and involves cash payments to poor families within Brazil, conditional on educational and health requirements [15]. Under BFP, cash benefits are preferentially paid to women in qualifying households. To be eligible, households must be registered in the Brazil's National Registry for Social Programmes "Cadastro Único" (CadUnico), and have a household income below a defined extreme poverty threshold (monthly per capita household income < BRL77 in 2014 (approximately USD19)) or poverty threshold (monthly per capita household income < BRL154 in 2014 (approximately USD39)). A number of changes to income thresholds for eligibility to BFP have occurred from 2004 onwards (Table 2). Extremely poor families receive a fixed benefit of BRL77 plus additional amounts for pregnant women, children and adolescents. Families defined as poor receive the supplements for pregnant women, children and any adolescents in the household, but not the fixed benefit. In the first years of BFP implementation, indigenous and quilombola communities (old African settlements) were prioritized to start receiving the benefit [15]. Receipt of benefit is conditional on families meeting certain qualifying conditions: pregnant women must access prenatal care; children aged 0-5 years and breastfeeding women must undergo monitoring by health professionals (vaccination and health check-ups at home or in the nearest primary healthcare centre); and school-age children must attend school for at least 85% of schooling days. If individuals do no longer meet the inclusion criteria, ie., if they improve their socioeconomic status, do not meet the conditionalities or do not update the registry every two years, the benefit will only continue for two more years.

For peer review only - http://bmjopen.bmj.com/site/about/guidelines.xhtml

Logic model

We created a logic model (Figure 1), informed by the existing literature, to describe the hypothesised mechanisms through which BFP may have an effect on CVD outcomes. We identified pathways that are likely to operate through material impacts, education and health conditionalities, and by fostering social inclusion, as well as distinguishing short, medium and long-term timescales. Possible short term impacts include changes in nutritional status in adults, through reduced salt and ultra-processed foods intake, increased consumption of foods with high nutrient content, and increases in overall energy intake [18-23]. BFP can also affect the socioeconomic conditions of the household, including more working hours, better jobs and higher income [24-26]. Effects of CCTs that may be observed at in the medium term include those intermediated by the health conditionalities of BFP, such as increased health care utilization [27-29]. Possible long term impacts include those stimulated by reductions in early life and cumulative socioeconomic adversity [30, 31], improved access to education and upward social mobility [32, 33]. Other potential impacts include the cumulative effects of better nutrition, reduced chronic stress and improved access to primary health services [19, 34-36], which can contribute to the control of hypertension, obesity and diabetes and longer survival following acute CVD events [37-39]. By providing an income primarily to women, and being accessible to disadvantaged ethno-racial groups, BFP may also contribute directly to reducing race and gender, as well as socio-economic, inequalities in CVD mortality [40-45].

Datasets

Our sample comprises members of families who applied for BFP or other social benefits from 2001 to 2015 registered in CadUnico, combined as part of the baseline of *The 100 Million Brazilian Cohort* [46]. We deterministically linked the baseline information of individuals from the 100 Million Brazilian Cohort (2001-2015) with BFP data (2004-2015) and used a semi-deterministic linkage based on five identifiers (name, sex, year of birth, name of the mother and municipality of residency) to link with individual level mortality records from the Mortality Information System (SIM) (2001-2015) [46]. From 2001 to 2015, over 114 million individuals applied for social benefits, but the final deidentified dataset will include information on the 92 million individuals that applied between 2004 and 2015 (Figure 2).

Sociodemographic variables

The baseline of the 100 Million Brazilian Cohort includes a range of sociodemographic variables collected at individuals' first application in CadÚnico, and includes household income, gender, age, race/ethnicity, geographical region and urban vs. rural, housing characteristics and education (Table 1). Completeness varies widely from variable to variable. Our cohort also contains baseline household monthly per capita income, which was calculated to reflect BFP eligibility criteria and comprises the sum of all household members' income from work, donations, pension and other benefits in the past month of registration, divided by the number of individuals living in the household [47]. For individuals that apply during or after 2011, the work component from the monthly per

capita income will be calculated as the lowest value of the total individual income from work in the past month or the sum of an individual's income from work in the past 12 months divided by 12.

Housing characteristics (such as number of rooms within the dwelling, building material used for construction, presence of indoor drinking water and sanitation) also provide a direct measure of material circumstances, which is less variable over time than income. Highest educational attainment reflects socioeconomic position in early adulthood, since for most people it remains constant after the age of 25 years. As is the case for most administrative datasets, there have been a number of changes in the data collection processes used by the BFP administrators. Changes in the collected variables were harmonized between the different versions when possible but kept separate when there were substantial differences between the studied years.

To account for changes in the eligibility criteria for BFP over time (Table 2), we will standardize the monthly per capita household income to the 2014 threshold. We will multiply the household per capita income value by 1.54 if individuals apply to BFP prior to 28th December 2007, by 1.283 if individuals apply to BFP between 28th December 2007 and 1st September 2009, and by 1.1 if they apply between 1st September 2009 and 31st May 2014 (See Table 2, column 5).

Bolsa Familia Program

The BFP data will provide information on the date each family member started and finished receiving BFP benefit from 1st January 2004 to 31st December 2015. For each individual we will include information on the first and last date they received BFP benefit.

Mortality Information System

Deaths within Brazil are subject to certification by medical professionals so the causes of death (using ICD-10 codes) can be ascertained reasonably precisely. Despite the significant and continuous improvement of data quality, regional disparities remain with the worst quality in the poorest regions and those with worse health care. In 2000, 14.3% of all deaths corresponded to ill-defined causes and this proportion varied from 28.4% in the Northeast to 6.3% in the South. Since 2005, the Ministry of Health has initiated several actions aimed at improving the quality of mortality information with an emphasis on the North and Northeast regions. By 2010, the proportion of ill-defined causes had dropped to 8.6%, but regional inequalities remained. Coding of CVD related causes is thought to be good, but for some analyses, corrections need to take into account ill-defined causes [48].

Data analysis plan

Data cleaning and preparation

The 100 Million Brazilian Cohort was already cleaned and variables were standardized according to strict protocols developed by Cidacs/Fiocruz. We will start by checking the linkage quality between the 100 Million Brazilian cohort and mortality, check data quality for obvious errors by inspecting the distribution of variables, will investigate the presence

For peer review only - http://bmjopen.bmj.com/site/about/guidelines.xhtml

of missing data, and make comparisons across similar variables to identify inconsistencies. We will also explicitly check for inconsistencies in data quality between geographical regions and over time. In the presence of substantial discrepancies between regions in the ascertainment of recording of cause of deaths, we shall stratify the analyses by region. Following initial checking and further data cleaning if necessary, we will develop derived variables harmonised over time and will maintain a detailed record and reproducible syntax for the process.

Definitions of exposures and outcomes

As BFP was first implemented in 2004, we will only include in our analysis individuals who registered to the 100 Million Brazilian cohort baseline on the 1st January 2004 onwards (Figure 2). Since many applicants will go on to receive BFP eventually, we will allocate exposure status based on whether they are a recipient within different time intervals following first application (Figure 3). Two approaches to classifying exposure status will include BFP recipients being defined as those who start receiving the benefit within 2 months and within 1 year after registering in the 100 Million Brazilian Cohort baseline.

To evaluate all-cause and cardiovascular mortality among individuals of all ages, the follow-up time (in years) for each individual will start when they first apply to 100 Million Brazilian cohort and will end at death by any cause or on 31st December 2015. To evaluate all-cause and cardiovascular premature death, follow-up time (in years) for each individual will start when they first apply to the 100 Million Brazilian cohort or, for individuals below 30 years old, on the date they complete 30 years of age. For this analysis, follow-up time will end at death, end of follow-up (31st December 2015), or on reaching 70 years of age.

Our primary outcome is premature cardiovascular disease mortality among adults (ICD codes I00-I99), defined as deaths occurring between 30 and 69 years of age. We shall also estimate models for all-ages and premature all-cause mortality to check the sensitivity of our overall approach to the analysis, and for subgroups of cardiovascular death, including ischaemic heart disease (I20-I25), and cerebrovascular disease (I60-I69) (see Figure 2).

Analysis

The final choice of methods for identifying the impact of BFP on CVD mortality will depend on initial exploratory analyses of the exposure and covariate data available in the 100 Million Cohort Dataset. We will stratify the analyses by year of application in the 100 Million Brazilian Cohort (<2010 and ≥2011) to reflect the changes in the income calculation in both periods, which is the main eligibility criteria for BFP receipt.

For individuals that apply ≥2011, in which we have better recorded income data (i.e. preliminary data cleaning showed >75% of individuals with monthly per capita income <BRL1 or <USD0.25 prior to 2011), one possibility is to use a regression discontinuity design (RDD). RDD uses a threshold in a continuous ('forcing') variable that determines exposure to an intervention to distinguish exposed and unexposed units. The key identifying assumption is that units within a narrow range of values ('bandwidth') of this

For peer review only - http://bmjopen.bmj.com/site/about/guidelines.xhtml

forcing variable either side of the threshold value will be similar in characteristics other than exposure, so that differences in outcomes can be interpreted as effects of the intervention.

Standardized monthly per capita household income will be used as the forcing variable, and we will conduct analysis for both the extreme poverty threshold (BRL77 / USD19 in 2004) and the poverty threshold (BRL154 / USD39 in 2004). Because eligibility for BFP depends partially on factors other than income, the probability of receipt will not change from 0 to 1 at the income threshold. We shall apply a fuzzy RDD, using two stage least squares, to model receipt of BFP as a function of income and the threshold in the first stage, and fitting the modelled probabilities of receipt as predictors in the outcome models.

We will estimate the effect of BFP on each of the study outcomes using Poisson or Cox proportional hazards survival models, accounting for the length of follow-up of each individual in the cohort[41, 49]. Since individuals are nested within a household, we will use multilevel models to account for the lack of independence of observations[41]. As BFP receipt may vary over time, we will define exposure status on the basis of first receipt. Once a household starts receiving BFP, individuals within the household will continue to be allocated to the exposed group (irrespective of subsequent changes to BFP status). Alternative approaches will include truncating the follow-up period to five years and excluding households that do not receive BFP for a minimum of five years and considering the treatment indicator as varying over time. We will stratify the analyses by year of application in the 100 Million Brazilian Cohort (<2010 and ≥2011) to reflect the changes in the income calculation in both periods. In addition, to test for differences in the effect of BFP by subgroup, we will stratify the analyses by geographical region (South, Southeast, Central-west, North and Northeast) and gender (male and female), household structure, highest educational attainment, housing conditions, urbanicity and municipality level deprivation measures.

One important problem with validity in studies using RDD is manipulation of assignment, for example by applicants understating their income. We shall plot histograms of income to test for smoothness at the thresholds, and scatterplots of covariates against income to check for any bunching below the thresholds that might indicate manipulation, and apply the McCrary test if there is any visual indication of manipulation[50].

We shall perform a series of robustness checks and sensitivity analyses[51]. We shall use plots of the outcomes against income to decide the most appropriate functional form. Given the large numbers available for analysis in the 100 Million Brazilian Cohort, we expect to be able to restrict the analyses to households within a narrow range of income either side of the threshold, and to estimate models within a range of bandwidths to test the robustness of the assumption of local linearity, to potentially increase the generalizability of our findings and to compare with further analysis including propensity score based methods. We will perform additional analysis restricted to a subgroup of individuals whose treatment has not varied over time and removing families with zero income to improve the robustness of our results. In addition, we will test the robustness

For peer review only - http://bmjopen.bmj.com/site/about/guidelines.xhtml

of our local effect estimates adjusting the survival models for age or other strong potential confounders. We shall also repeat the analyses with data limited to the most reliable data available, e.g. excluding geographical areas where data quality is poor and therefore a high percentage of garbage codes and missing mortality data are expected.

Aside from RDD, separately for individuals that applied <2010 and ≥2011, we will estimate the effect of BFP on all-cause and cardiovascular mortality using propensity score-based methods. Propensity score methods seek to reduce confounding through the comparison of units that have similar probabilities to be exposed or unexposed to the intervention, given observed characteristics[52]. We will use risk-set matching to calculate the probability of receiving BFP over time, given the baseline demographic and socioeconomic variables of the 100 Million Cohort[53, 54]. We will match BFP and non-BFP participants overall or within strata using the propensity score. Matching methods will include nearest neighbour matching using adequate calliper and replacement. We will estimate the average treatment effect on the treated (ATT) of BFP on overall mortality and cardiovascular outcomes using Cox or Poisson models.

To verify the hypothesis that matching could be pruning similar individuals, we will estimate in the unmatched cohort the ATT and the average treatment effect (ATE) using survival models weighted by the inverse of the probability of receiving the treatment (inverse probability of the treatment weighting - IPTW). Also, we will estimate both ATT and ATE in quintiles or deciles of propensity score strata. For all the methods that rely on observable covariates, we will check balance of covariates between the intervention and control groups.

Our preference, as informed by the MRC's guidance on the evaluation of natural experiments [45], is to use both RDD and propensity score based methods, since each method relies on different assumptions (e.g. no unobserved confounding for propensity score based methods and as-if random allocation at the eligibility cut-off for BFP in the case of RDD). Obtaining consistent results from the two approaches for individuals that applied ≥2011 will, along with the other robustness checks, strengthen confidence in our inferences about impact. For each of the analyses we will evaluate whether the assumptions of the corresponding methodology are tenable. The exploratory analyses and decisions leading to the final specification of the analyses will be fully documented and reported alongside the findings.

Effect modification and intersectionality

We will investigate differential impacts of BFP on mortality by including interaction terms within the models and by stratifying the analyses to provide estimates for population subgroups defined by gender, race/ethnicity, urbanicity and socioeconomic position (education and area-level deprivation). To investigate intersectionality, we will test for effect measure modification on both an additive scale and a multiplicative scale [55, 56]. We are especially interested in exploring variation according to combinations of characteristics, such as gender and race/ethnicity (Figure 4) [55]. Therefore, we will create a single categorical variable that incorporates the two concepts of interest and estimate the relative excess risk due to interaction [56]. To study intersectionality on a

For peer review only - http://bmjopen.bmj.com/site/about/guidelines.xhtml

multiplicative scale, we will fit interaction terms between variables representing the two concepts. For further subgroup analyses, we shall submit a full specification of the analysis of interest to the SPHI project steering group before data analysis begins and report the results of all analyses rather than selecting according to size or significance of effects.

Ethics and dissemination

The 100 Million Brazilian Cohort study was approved by the ethics committee of Gonçalo Muniz Institute - Oswaldo Cruz Foundation (1.612.302) and the specific aims of this project were submitted for ethical approval to the same ethics committee. The University of Glasgow Medical, Veterinary & Life Sciences College Ethics Committee also approved the study (project number: 200190001). All data are linked in a safe room with access to restricted people only. After data are linked and linkage accuracy is calculated, researchers will have full access to the deidentified dataset. The dataset will be accessed by researchers upon application to a data curation committee with a detailed analysis plan. The dataset will receive a Digital Object Identifier System (DOI), and full specification of how the dataset was created will be available online. All manuscripts will be published in open access journals. The study is part of a larger project, the NIHR Global Health Research Group on Social Policy and Health Inequalities, which supports a communications group responsible for producing summaries of the published research results for managers, policymakers and the broader public.

Author contributions: JP, PC, EA and SVK wrote the first draft of the protocol. MA, LA, SA, LS, MLB and AL contributed with additional material. All authors participated with the discussions during the development of this protocol and reviewed the final version.

Funding statement: This research was funded by the National Institute for Health Research (NIHR) (GHRG /16/137/99) using UK aid from the UK Government to support global health research. The views expressed in this publication are those of the author(s) and not necessarily those of the NIHR or the UK Department of Health and Social Care. The Social and Public Health Sciences Unit is core funded by the Medical Research Council (MC_UU_12017/13) and the Scottish Government Chief Scientist Office (SPHSU13). SVK is funded by a NHS Research Scotland Senior Clinical Fellowship (SCAF/15/02). Cidacs/Fiocruz is supported by grants from CNPq/MS/Gates Foundation (401739/2015-5) and the Wellcome Trust, UK (202912/Z/16/Z).

Competing interests statement: The authors declare no competing interests.

References

1. Bastagli F. Poverty, inequality and public cash transfers: lessons from Latin America. 2010.

2. Ivaschenko O, Alas CPR, Novikova M, Romero C, Bowen T, Chu LJ. The State of Social Safety Nets 2018. Washington: The World Bank; 2018.

For peer review only - http://bmjopen.bmj.com/site/about/guidelines.xhtml

3. Bastagli F, Hagen-Zanker J, Harman L, Barca V, Sturge G, Schmidt T. The Impact of Cash Transfers: A Review of the Evidence from Low- and Middle-income Countries. Journal of Social Policy. 2019;48(3):569-94. doi: 10.1017/s0047279418000715.
4. Fiszbein A, Schady NR. Conditional cash transfers: reducing present and future poverty: The World Bank; 2009.
5. Gloria M, Laura B. Evaluating the impact of conditional cash transfer programs: lessons from Latin America. Policy Research Working Paper Series. 2003.
6. PAHO. World Heart Day: Cardiovascular Diseases Cause 1.9 Million Deaths a Year in the Americas: Pan American Health Organization (PAHO); 2019 [cited 2019 06/08//2019]. Available from: https://www.paho.org/.
7. GBD Compare [Internet]. IHME, University of Washington. 2019 [cited 24/09/2019]. Available from: http://vizhub.healthdata.org/gbd-compare.
8. Stringhini S, Carmeli C, Jokela M, Avendaño M, Muennig P, Guida F, et al. Socioeconomic status and the 25× 25 risk factors as determinants of premature mortality: a multicohort study and meta-analysis of 1· 7 million men and women. The Lancet. 2017;389(10075):1229-37.
9. Ordunez P, Prieto-Lara E, Pinheiro Gawryszewski V, Hennis AJM, Cooper RS. Premature Mortality from Cardiovascular Disease in the Americas – Will the Goal of a Decline of "25% by 2025" be Met? PLOS ONE. 2015;10(10):e0141685. doi: 10.1371/journal.pone.0141685.
10. Paiva L. Poverty and Inequality Reduction in Brazil–A Parenthesis in History or the Road Ahead? Iberoamericana–Nordic Journal of Latin American and Caribbean Studies. 2016;45(1).
11. Marinho F, de Azeredo Passos VM, Malta DC, França EB, Abreu DM, Araújo VE, et al. Burden of disease in Brazil, 1990–2016: a systematic subnational analysis for the Global Burden of Disease Study 2016. The Lancet. 2018;392(10149):760-75.
12. Brant LCC, Nascimento BR, Passos VMA, Duncan BB, Bensenõr IJM, Malta DC, et al. Variações e diferenciais da mortalidade por doença cardiovascular no Brasil e em seus estados, em 1990 e 2015: estimativas do Estudo Carga Global de Doença. Revista Brasileira de Epidemiologia. 2017;20:116-28.
13. Barros MBdA, Lima MG, Medina LdPB, Szwarcwald CL, Malta DC. Social inequalities in health behaviors among Brazilian adults: National Health Survey, 2013. International journal for equity in health. 2016;15(1):148-. doi: 10.1186/s12939-016-0439-0. PubMed PMID: 27852275.
14. Soares S, De Souza PHGF, Osório RG, Silveira FG. Os impactos do benefício do Programa Bolsa Família sobre a desigualdade e a pobreza. In: IPEA, editor. Bolsa Família 2003-2010: avanços e desafios. 2010. Brasilia2010. p. 27-52.
15. Campello T, Neri MC. Programa Bolsa Família: uma década de inclusão e cidadania. Brasília: Ipea. 2013.
16. Brasil, MDS. Relatórios de Informações Sociais - Bolsa Família e Cadastro Único: Ministerio da Cidadania (MDS); 2020 [cited 2020 31/03/2020]. Available from: https://aplicacoes.mds.gov.br/sagi/RIv3/geral/index.php?relatorio=153&file=entrada#.
17. Campbell M, Katikireddi SV, Hoffmann T, Armstrong R, Waters E, Craig P. TIDieR-PHP: a reporting guideline for population health and policy interventions. bmj. 2018;361:k1079.
18. Coelho PL, Melo A. The impact of the "Bolsa Familia" Program on household diet quality, Pernambuco State, Brazil. Cienc Saude Coletiva. 2017;22(2):393-402. doi: 10.1590/1413-81232017222.13622015. PubMed PMID: WOS:000394147100007.
19. Martins APB, Monteiro CA. Impact of the Bolsa Família program on food availability of low-income Brazilian families: A quasi experimental study. BMC Public Health. 2016;16(1). doi: 10.1186/s12889-016-3486-y.
20. Ferrario MN. The impacts on family consumption of the Bolsa Familia subsidy programme. Cepal Rev. 2014;(112):147-63. PubMed PMID: WOS:000340076200008.

For peer review only - http://bmjopen.bmj.com/site/about/guidelines.xhtml

21. Sperandio N, Rodrigues CT, Franceschini SdCC, Priore SE. Impacto do Programa Bolsa Família no consumo de alimentos: estudo comparativo das regiões Sudeste e Nordeste do Brasil. Ciênc Saúde Colet. 2017;22(6):1771-80.
22. Sperandio N, Rodrigues CT, Franceschini SDC, Priore SE. Impact of the Bolsa Familia Program on energy, macronutrient, and micronutrient intakes: Study of the Northeast and Southeast. Rev Nutr. 2016;29(6):833-44. doi: 10.1590/1678-98652016000600008. PubMed PMID: WOS:000391443800009.
23. Baptistella JCF. Avaliação de programas sociais: uma análise do impacto do Bolsa Família sobre o consumo de alimentos. 2012.
24. Cabral CS, Lopes AG, Lopes JM, Vianna RPT. Food security, income, and the Bolsa Família program: A cohort study of municipalities in Paraíba State, Brazil, 2005-2011. Cad Saude Publica. 2014;30(2):393-402. doi: 10.1590/0102-311X00140112.
25. Barbosa ALNH, Corseuil CHL. Conditional cash transfer and informality in Brazil. IZA J Labor Dev. 2014;3(1). doi: 10.1186/s40175-014-0024-0.
26. Nazareno LdA. O programa bolsa família e o mercado de trabalho informal: Uma análise de impacto da ação Brasil carinhoso. Econ Apl. 2016;20(4):457-71. doi: 10.11606/1413-8050/ea153891.
27. Fernald LC, Gertler PJ, Hou X. Cash component of conditional cash transfer program is associated with higher body mass index and blood pressure in adults. The Journal of nutrition. 2008;138(11):2250-7.
28. Gertler P. The impact of PROGRESA on health. 2000 2000. Report No.
29. Witvorapong N, Foshanji AI. The impact of a conditional cash transfer program on the utilization of non-targeted services: Evidence from Afghanistan. Social Science & Medicine. 2016;152:87-95.
30. Pollitt RA, Rose KM, Kaufman JS. Evaluating the evidence for models of life course socioeconomic factors and cardiovascular outcomes: a systematic review. BMC public health. 2005;5(1):7.
31. Hardy R, Lawlor DA, Kuh D. A life course approach to cardiovascular aging. Future cardiology. 2015;11(1):101-13.
32. de Brauw A, Gilligan DO, Hoddinott J, Roy S. The Impact of Bolsa Familia on Schooling. World Development. 2015;70:303-16. doi: 10.1016/j.worlddev.2015.02.001. PubMed PMID: WOS:000351969500023.
33. Glewwe P, Kassouf AL. The impact of the Bolsa Escola/Familia conditional cash transfer program on enrollment, dropout rates and grade promotion in Brazil. J Dev Econ. 2012;97(2):505-17. doi: 10.1016/j.jdeveco.2011.05.008.
34. Simões AA, Sabates R. The Contribution of Bolsa Família to the educational achievement of economically disadvantaged children in Brazil. Int J Educ Dev. 2014;39:141-56.
35. Santos TMd, Silva SSdC, Koller SH. Evaluation of Amazon Riverine Beneficiaries about Bolsa Família Program. Psicologia: Teoria e Pesquisa. 2017;33.
36. Shei A, Costa F, Reis MG, Ko AI. The impact of Brazil's Bolsa Família conditional cash transfer program on children's health care utilization and health outcomes. BMC Int Health Hum Rights. 2014;14(1). doi: 10.1186/1472-698X-14-10.
37. Chandola T, Britton A, Brunner E, Hemingway H, Malik M, Kumari M, et al. Work stress and coronary heart disease: what are the mechanisms? European heart journal. 2008;29(5):640-8. Epub 2008/01/25. doi: 10.1093/eurheartj/ehm584. PubMed PMID: 18216031.
38. Chandola T, Brunner E, Marmot M. Chronic stress at work and the metabolic syndrome: prospective study. Bmj. 2006;332(7540):521-5. Epub 2006/01/24. doi: 10.1136/bmj.38693.435301.80. PubMed PMID: 16428252; PubMed Central PMCID: PMCPMC1388129.
39. Hjelm L, Handa S, de Hoop J, Palermo T. Poverty and perceived stress: Evidence from two unconditional cash transfer programs in Zambia. Social science & medicine (1982).

For peer review only - http://bmjopen.bmj.com/site/about/guidelines.xhtml

2017;177:110-7. Epub 2017/02/09. doi: 10.1016/j.socscimed.2017.01.023. PubMed PMID: 28167339.
40. Attanasio O, Battistin E, Mesnard A. Food and Cash Transfers: Evidence from Colombia. The Economic Journal. 2012;122(559):92-124. doi: 10.1111/j.1468-0297.2011.02473.x.
41. Tommasi D. Control of resources, bargaining power and the demand of food: Evidence from PROGRESA. Journal of Economic Behavior & Organization. 2019;161:265-86.
42. Brauw A, Gilligan DO, Hoddinott J, Roy S. The Impact of Bolsa Famı´lia on Women's Decision-Making Power. World Development. 2014;59:487-504. doi: 10.1016/j.worlddev.2013.02.003.
43. Carloto C, Mariano SA. Empoderamento, trabalho e cuidados: mulheres no programa bolsa família. Textos & Contextos (Porto Alegre). 2012;11(2).
44. Bell CN, Thorpe RJ, Jr., Bowie JV, LaVeist TA. Race disparities in cardiovascular disease risk factors within socioeconomic status strata. Annals of epidemiology. 2018;28(3):147-52. Epub 2018/01/11. doi: 10.1016/j.annepidem.2017.12.007. PubMed PMID: 29317176.
45. Brondolo E. Considering social processes when examining mediators of the effects of discrimination on health. Social Science & Medicine. 2018;215:160-2. doi: https://doi.org/10.1016/j.socscimed.2018.08.004.
46. Ali MS, Ichihara MY, Lopes LC, Barbosa GCG, Pita R, Carreiro RP, et al. Administrative Data Linkage in Brazil: Potentials for Health Technology Assessment. Front Pharmacol. 2019;10:984-. doi: 10.3389/fphar.2019.00984. PubMed PMID: 31607900.
47. Brasil. Perguntas Frequentes sobre Dados do Cadastro Único para Programas Sociais. In: (Senarc) MdDSMSNdRdC, editor. Brasilia2011.
48. França E, Teixeira R, Ishitani L, Duncan BB, Cortez-Escalante JJ, Morais Neto OLd, et al. Ill-defined causes of death in Brazil: a redistribution method based on the investigation of such causes. Rev Saude Publica. 2014;48:671-81.
49. Tchetgen EJT, Walter S, Vansteelandt S, Martinussen T, Glymour M. Instrumental variable estimation in a survival context. Epidemiology (Cambridge, Mass). 2015;26(3):402.
50. Imbens GW, Lemieux T. Regression discontinuity designs: A guide to practice. Journal of econometrics. 2008;142(2):615-35.
51. Lee DS, Lemieux T. Regression discontinuity designs in economics. Journal of economic literature. 2010;48(2):281-355.
52. Rubin DB. Estimating causal effects from large data sets using propensity scores. Annals of internal medicine. 1997;127(8_Part_2):757-63.
53. Watson D, Spaulding AB, Dreyfus J. Risk-set matching to assess the impact of hospital-acquired bloodstream infections. American journal of epidemiology. 2019;188(2):461-6.
54. Danaei G, Rodríguez LAG, Cantero OF, Logan RW, Hernán MA. Electronic medical records can be used to emulate target trials of sustained treatment strategies. Journal of clinical epidemiology. 2018;96:12-22.
55. Bauer GR. Incorporating intersectionality theory into population health research methodology: challenges and the potential to advance health equity. Social science & medicine. 2014;110:10-7.
56. Knol MJ, VanderWeele TJ. Recommendations for presenting analyses of effect modification and interaction. International journal of epidemiology. 2012;41(2):514-20.

For peer review only - http://bmjopen.bmj.com/site/about/guidelines.xhtml

TABLES AND FIGURES

Table 1. Key variables available for the analysis from the 100 Million Brazilian Cohort baseline, from Bolsa Familia dataset and from mortality data.

100 Million Brazilian Cohort baseline	
Individual level variables	
**Age (at application)**	Continuous
**Sex**	Female, Male
**Relationship with the responsible person for the household**	Responsible him/herself, wife/husband, son/daughter, stepson/stepdaughter, grandchildren, parent-in-law, brother/sister, son/daughter-in-law, other relative, not relative.
**Race/Ethnicity**	White, Brown, Black, Asian, Indigenous
**Literacy**	Literate or illiterate.
**Level of education**	Never went to school, Pre-school, literacy school, primary education (first stage - 5 years), primary education (second stage - 4 years), High School, Higher education
**Monthly per capita income[1]**	Sum of income from work[2], donations, pension and others per divided by the number of individuals in the household in the given year.
Family level variables	
**Municipality of family home**	Single identifier for every municipality
**Region of family home**	South, South-east, North, North-east, Central-West
**Location of family home**	Urban, rural
**Housing material**	Brick or cement, Taipa, Wood, Other
**Household type**	Private, improvised but private, collective, others.
**Household water supply**	Public network, Well or natural source, cistern or others
**Sewage disposal system**	Public network, Septic tank, rudimentary tank, ditch, others.
**Electricity**	Home meter, Community meter, irregular electricity, gas lighting, candlelight, other
**Waste collection**	Public collection system, burned, buried, outdoor disposal, other
**Number of individuals in the household**	Continuous
**Number of rooms in the household (including bathrooms, living room and kitchen if separated by walls)**	Continuous
Bolsa Familia Program variables	
**Benefit starting date**	Date
**Benefit ending date**	Date
**Duration of the benefit receipt**	Time in years
Mortality system information (SIM)	
**Date of death**	Date
**Place of death**	Hospital, other health establishments, household, street, others
**Municipality of death**	Single identifier for every municipality
**Medical assistance**	Yes or No

For peer review only - http://bmjopen.bmj.com/site/about/guidelines.xhtml

**Necropsy investigation**	Yes or No
**Main cause of death**	ICD-10 categories

Table 2. Eligibility criteria for Bolsa Familia Program (BFP) receipt in Brazil between 2004 and 2015.

Year	Extremely poverty eligibility criteria[1]	Poverty eligibility criteria[1]	Date of change	Standardization rate[2] from 2004 to 2014 thresholds
2004	BRL 50 (USD12.5)	BRL 100 BRL (USD26)		1.540
2007	BRL 60 (USD15)	BRL 120 BRL (USD30)	28th Dec 2007	1.283
2009	BRL 70 (USD17.5)	BRL 140 BRL (USD35)	1st Sept 2009	1.100
2014	BRL 77 (USD19.3)	BRL 154 BRL (USD38.5)	1st Jun 2014	-

[1]Values in USD were calculated based on the following exchange rate: 4BRL=1USD.

[2]For each year (2004, 2007 and 2009), the standardized rate was estimated by dividing the poverty (or extreme poverty) criteria from 2014 by the poverty criteria (or extreme poverty) from that year.

For peer review only

For peer review only - http://bmjopen.bmj.com/site/about/guidelines.xhtml

Table 3. All cause and cardiovascular mortality rates among individuals from the 100 Million Brazilian Cohort (N=92 Million).

	Deaths (x10³)	Mean yearly mortality rates per 100,000 person-years at risk during the study period		
		**Overall**	**Male**	**Female**
**Overall population (N=92 millions)**				
All cause mortality rate	1,810	286	370	199
Cardiovascular mortality				
All cardiovascular mortality rate (I00-99)	432	68	81	56
Cerebrovascular mortality rate (I60-69)	113	18	20	15
Ischaemic heart diseases mortality rate (I20-25)	117	19	24	13
**Individuals aged 30-69 y (N=34 millions)**				
All cause premature mortality rate	943	482	604	344
Premature cardiovascular mortality				
CVD premature mortality rate (I00-99)	243	124	147	98
Cerebrovascular premature mortality rate (I60-69)	63	32	36	28
Ischaemic heart diseases premature mortality rate (I20-25)	76	39	50	26

For peer review only

Fig 1. Logic Model of the potential effect of Bolsa Familia Program (BFP) on all-cause and cardiovascular mortality.

Fig 2. Flowchart of data selection.

Fig 3. Approximate cumulative number and proportion of individuals of all ages receiving BFP over time after start of follow-up for those that apply between 2004-2010 (Cohort 1) (A) and for those that apply between 2011-2015 (Cohort 2) (B).

Fig 4. Main strata to investigate intersectionality regarding gender and race/ethnicity among individuals from all ages from the 100 Million Brazilian Cohort (2004-2015).

For peer review only

For peer review only - http://bmjopen.bmj.com/site/about/guidelines.xhtml

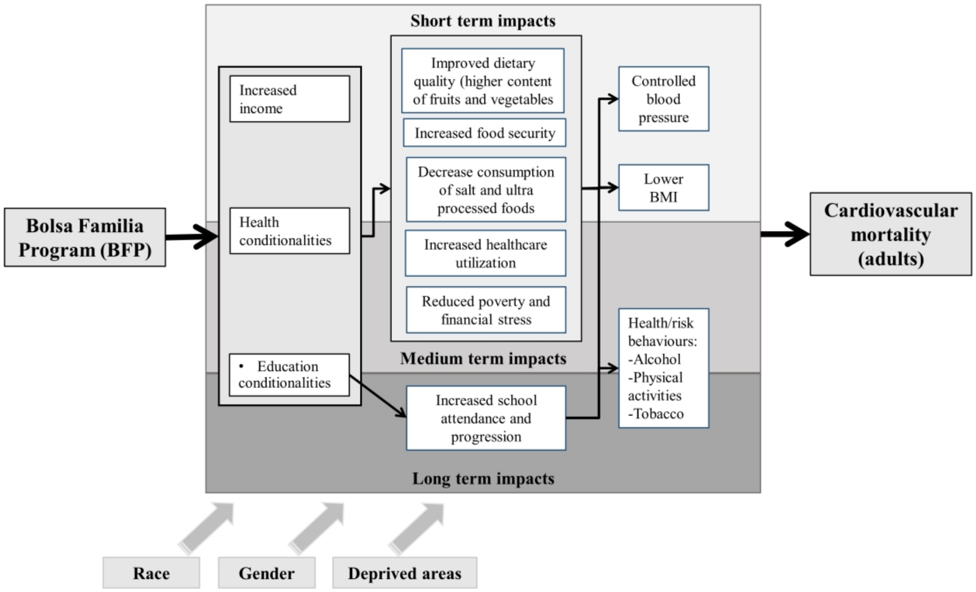

Fig 1. Logic Model of the potential effect of Bolsa Familia Program (BFP) on all-cause and cardiovascular mortality.

279x176mm (300 x 300 DPI)

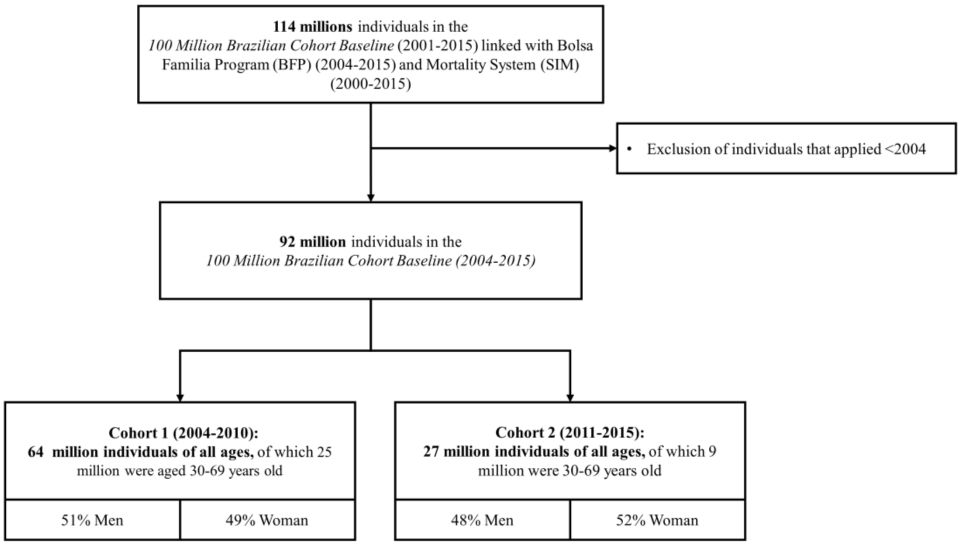

Fig 2. Flowchart of data selection.

270x170mm (300 x 300 DPI)

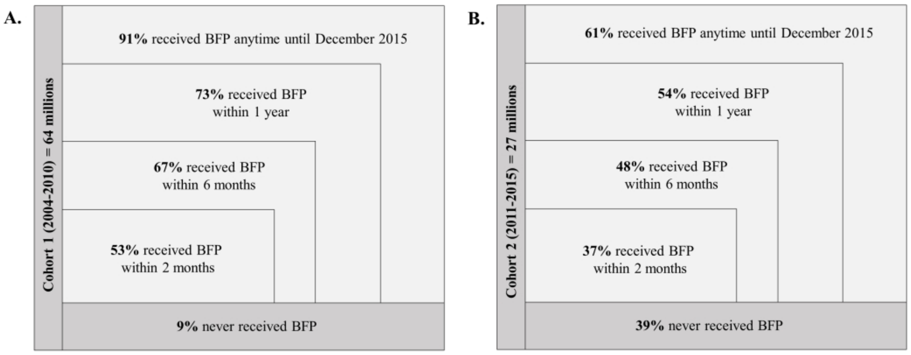

Fig 3. Approximate cumulative number and proportion of individuals of all ages receiving BFP over time after start of follow-up for those that apply between 2004-2010 (Cohort 1) (A) and for those that apply between 2011-2015 (Cohort 2) (B).

338x190mm (300 x 300 DPI)

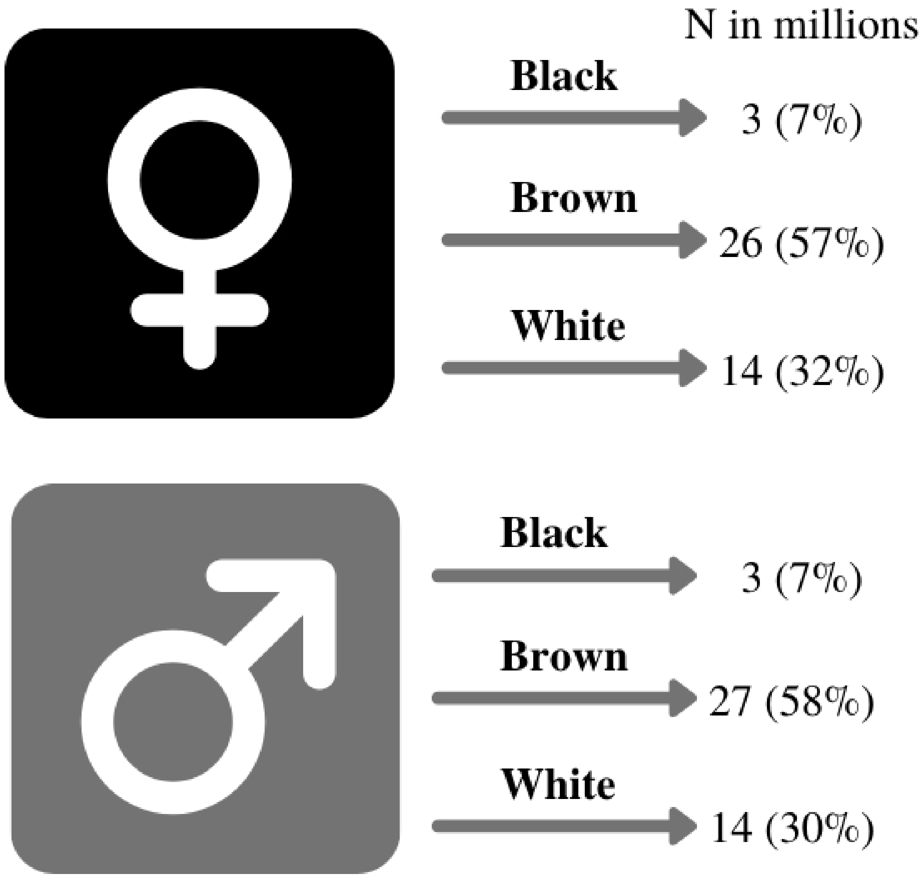

Fig 4. Main strata to investigate intersectionality regarding gender and race/ethnicity among individuals from all ages from the 100 Million Brazilian Cohort (2004-2015).

STROBE Statement—checklist of items that should be included in reports of observational studies

	Item No.	Recommendation	Page No.	Relevant text from manuscript
**Title and abstract**	1	(*a*) Indicate the study's design with a commonly used term in the title or the abstract	1;2	
		(*b*) Provide in the abstract an informative and balanced summary of what was done and what was found	2	
**Introduction**				
Background/rationale	2	Explain the scientific background and rationale for the investigation being reported	4	
Objectives	3	State specific objectives, including any prespecified hypotheses	4-6	
**Methods**				
Study design	4	Present key elements of study design early in the paper	5	
Setting	5	Describe the setting, locations, and relevant dates, including periods of recruitment, exposure, follow-up, and data collection	6	
Participants	6	(*a*) *Cohort study*—Give the eligibility criteria, and the sources and methods of selection of participants. Describe methods of follow-up<br>*Case-control study*—Give the eligibility criteria, and the sources and methods of case ascertainment and control selection. Give the rationale for the choice of cases and controls<br>*Cross-sectional study*—Give the eligibility criteria, and the sources and methods of selection of participants		
		(*b*) *Cohort study*—For matched studies, give matching criteria and number of exposed and unexposed<br>*Case-control study*—For matched studies, give matching criteria and the number of controls per case	6-10	
Variables	7	Clearly define all outcomes, exposures, predictors, potential confounders, and effect modifiers. Give diagnostic criteria, if applicable	8-10	
Data sources/ measurement	8*	For each variable of interest, give sources of data and details of methods of assessment (measurement). Describe comparability of assessment methods if there is more than one group	6-7	
Bias	9	Describe any efforts to address potential sources of bias	9-11	
Study size	10	Explain how the study size was arrived at	6	

Continued on next page

For peer review only - http://bmjopen.bmj.com/site/about/guidelines.xhtml

Quantitative variables	11	Explain how quantitative variables were handled in the analyses. If applicable, describe which groupings were chosen and why	NA
Statistical methods	12	(*a*) Describe all statistical methods, including those used to control for confounding	8-11
		(*b*) Describe any methods used to examine subgroups and interactions	9-11
		(*c*) Explain how missing data were addressed	10
		(*d*) *Cohort study*—If applicable, explain how loss to follow-up was addressed<br>*Case-control study*—If applicable, explain how matching of cases and controls was addressed<br>*Cross-sectional study*—If applicable, describe analytical methods taking account of sampling strategy	
		(*e*) Describe any sensitivity analyses	9-11
**Results**			
Participants	13*	(a) Report numbers of individuals at each stage of study—eg numbers potentially eligible, examined for eligibility, confirmed eligible, included in the study, completing follow-up, and analysed	NA
		(b) Give reasons for non-participation at each stage	NA
		(c) Consider use of a flow diagram	NA
Descriptive data	14*	(a) Give characteristics of study participants (eg demographic, clinical, social) and information on exposures and potential confounders	NA
		(b) Indicate number of participants with missing data for each variable of interest	NA
		(c) *Cohort study*—Summarise follow-up time (eg, average and total amount)	NA
Outcome data	15*	*Cohort study*—Report numbers of outcome events or summary measures over time	NA
		Case-control study—Report numbers in each exposure category, or summary measures of exposure	NA
		Cross-sectional study—Report numbers of outcome events or summary measures	NA
Main results	16	(*a*) Give unadjusted estimates and, if applicable, confounder-adjusted estimates and their precision (eg, 95% confidence interval). Make clear which confounders were adjusted for and why they were included	NA
		(*b*) Report category boundaries when continuous variables were categorized	NA
		(*c*) If relevant, consider translating estimates of relative risk into absolute risk for a meaningful time period	NA

Continued on next page

For peer review only - http://bmjopen.bmj.com/site/about/guidelines.xhtml

Other analyses	17	Report other analyses done—eg analyses of subgroups and interactions, and sensitivity analyses	NA
**Discussion**			
Key results	18	Summarise key results with reference to study objectives	NA
Limitations	19	Discuss limitations of the study, taking into account sources of potential bias or imprecision. Discuss both direction and magnitude of any potential bias	3
Interpretation	20	Give a cautious overall interpretation of results considering objectives, limitations, multiplicity of analyses, results from similar studies, and other relevant evidence	NA
Generalisability	21	Discuss the generalisability (external validity) of the study results	NA
**Other information**			
Funding	22	Give the source of funding and the role of the funders for the present study and, if applicable, for the original study on which the present article is based	11

*Give information separately for cases and controls in case-control studies and, if applicable, for exposed and unexposed groups in cohort and cross-sectional studies.

Note: An Explanation and Elaboration article discusses each checklist item and gives methodological background and published examples of transparent reporting. The STROBE checklist is best used in conjunction with this article (freely available on the Web sites of PLoS Medicine at http://www.plosmedicine.org/, Annals of Internal Medicine at http://www.annals.org/, and Epidemiology at http://www.epidem.com/). Information on the STROBE Initiative is available at www.strobe-statement.org.

For peer review only

BMJ Open

Evaluating the impact of the Bolsa Familia conditional cash transfer program on premature cardiovascular and all-cause mortality using the 100 million Brazilian cohort: A natural experiment study protocol

Journal:	*BMJ Open*
Manuscript ID	bmjopen-2020-039658.R1
Article Type:	Protocol
Date Submitted by the Author:	30-Jul-2020
Complete List of Authors:	Pescarini, Julia; Fundação Oswaldo Cruz, Centro de Integração de Dados e Conhecimentos para Saúde (Cidacs)<br>Craig , Peter ; University of Glasgow, MRC/CSO Social & Public Health Sciences Unit<br>Allik, Mirjam; University of Glasgow, MRC/CSO Social & Public Health Sciences Unit<br>Amorim, Leila; Universidade Federal da Bahia, Instituto de Matemática e Estatística<br>Ali, Sanni; London School of Hygiene & Tropical Medicine<br>Smeeth, Liam; London School of Hygiene & Tropical Medicine, Epidemiology and Population Health<br>Barreto, Mauricio; Universidade Federal da Bahia, Instituto de Saúde Coletiva; Fundação Oswaldo Cruz, Centro de Integração de Dados e Conhecimentos para Saúde (Cidacs)<br>Leyland, Alastair; University of Glasgow, MRC/CSO Social & Public Health Sciences Unit<br>Aquino, Estela M. L.; Universidade Federal da Bahia, Instituto de Saúde Coletiva<br>Katikireddi, Srinivasa; University of Glasgow School of Life Sciences, MRC/CSO Social & Public Health Sciences Unit
**Primary Subject Heading**:	Public health
Secondary Subject Heading:	Health policy, Epidemiology
Keywords:	Health policy < HEALTH SERVICES ADMINISTRATION & MANAGEMENT, PUBLIC HEALTH, Cardiac Epidemiology < CARDIOLOGY, EPIDEMIOLOGY

SCHOLARONE™
Manuscripts

I, the Submitting Author has the right to grant and does grant on behalf of all authors of the Work (as defined in the below author licence), an exclusive licence and/or a non-exclusive licence for contributions from authors who are: i) UK Crown employees; ii) where BMJ has agreed a CC-BY licence shall apply, and/or iii) in accordance with the terms applicable for US Federal Government officers or employees acting as part of their official duties; on a worldwide, perpetual, irrevocable, royalty-free basis to BMJ Publishing Group Ltd ("BMJ") its licensees and where the relevant Journal is co-owned by BMJ to the co-owners of the Journal, to publish the Work in this journal and any other BMJ products and to exploit all rights, as set out in our licence.

The Submitting Author accepts and understands that any supply made under these terms is made by BMJ to the Submitting Author unless you are acting as an employee on behalf of your employer or a postgraduate student of an affiliated institution which is paying any applicable article publishing charge ("APC") for Open Access articles. Where the Submitting Author wishes to make the Work available on an Open Access basis (and intends to pay the relevant APC), the terms of reuse of such Open Access shall be governed by a Creative Commons licence – details of these licences and which Creative Commons licence will apply to this Work are set out in our licence referred to above.

Other than as permitted in any relevant BMJ Author's Self Archiving Policies, I confirm this Work has not been accepted for publication elsewhere, is not being considered for publication elsewhere and does not duplicate material already published. I confirm all authors consent to publication of this Work and authorise the granting of this licence.

For peer review only

Evaluating the impact of the Bolsa Familia conditional cash transfer program on premature cardiovascular and all-cause mortality using the 100 million Brazilian cohort: A natural experiment study protocol

Julia M Pescarini[1*], Peter Craig[2], Mirjam Allik[2], Leila Amorim[3], Sanni Ali[4], Liam Smeeth[4,5], Mauricio L Barreto[1], Alastair H Leyland[2], Estela ML Aquino[6**], Srinivasa Vittal Katikireddi[2**].

1. Centro de Integração de Dados e Conhecimentos para Saúde (Cidacs), Fundação Oswaldo Cruz, Salvador, Brazil.
2. MRC/CSO Social & Public Health Sciences Unit, University of Glasgow, Glasgow, United Kingdom.
3. Instituto de Matemática e Estatística, Universidade Federal da Bahia, Salvador, Brazil.
4. Department of Non-Communicable Disease Epidemiology, London School of Hygiene & Tropical Medicine, London, United Kingdom.
5. Health Data Research (HDR), London, United Kingdom.
6. Instituto de Saúde Coletiva, Universidade Federal da Bahia, Salvador, Brazil.

***Corresponding Author:** Julia M. Pescarini
E-mail: juliapescarini@gmail.com (JMP)
Postal address: Centro de Integração de Dados e Conhecimentos para Saúde (Cidacs), Fundação Oswaldo Cruz. Edf. Tecnocentro – R. Mundo, 121 – Trobogy, Salvador – Bahia/Brazil. Postal code: 41745-715.
Telephone: +71 982608344

****Co-senior authors**

Word Count: (3,975 words)

Keywords: Cardiovascular diseases, quasi-experiment, social protection, social policies, cash-transfers.

For peer review only - http://bmjopen.bmj.com/site/about/guidelines.xhtml

Abstract

Introduction

Brazil's Bolsa Familia Programme (BFP) is the world's largest conditional cash transfer scheme. We shall use a large cohort of applicants for different social programmes to evaluate the effect of BFP receipt on premature all-cause and cardiovascular mortality.

Methods and analysis

We will identify BFP-recipients and non-recipients among new applicants from 2004-2015 in the 100 Million Brazilian Cohort, a database of 114 million individuals containing socio-demographic and mortality information of applicants to any Brazilian social programme. For individuals applying from 2011, when we have better recorded income data, we shall compare premature (age 30-69) cardiovascular and all-cause mortality among BFP-recipients and non-recipients using regression discontinuity design (RDD) with household monthly per capita income as the forcing variable. Effects will be estimated using survival models accounting for individual follow-up. To test the sensitivity of our findings, we will estimate models with different bandwidths, include potential confounders as covariates in the survival models, and restrict our data to locations with the most reliable data. In addition, we will estimate the effect of BFP on studied outcomes using propensity score risk-set matching, separately for individuals that applied ≤2010 and >2011, allowing comparability with RDD. Analyses will be stratified by geographical region, gender, race/ethnicity and socioeconomic position. We will investigate differential impacts of BFP and the presence of effect modification for a combination of characteristics, including gender and race/ethnicity.

Ethics and dissemination

The study was approved by the ethics committees of Oswaldo Cruz Foundation and the University of Glasgow College of Medicine and Veterinary Life Sciences. The deidentified dataset will be provided to researchers, and data analysis will be performed in a safe computational environment without internet access. Study findings will be published in high quality peer-reviewed research articles. The published results will be disseminated in the social media and to policy makers.

For peer review only - http://bmjopen.bmj.com/site/about/guidelines.xhtml

Article Summary

Strengths and limitations of this study

- Few previous studies of conditional cash-transfer programs have investigated impacts on adult health, or on premature death by CVD, using individual level data on exposures and outcomes.
- We exploit a nationwide linkage of social policy and health datasets to evaluate the largest CCT in the world in one of the most unequal countries.
- We use natural experimental approaches to estimate the effect of BFP that control for both observed and unobserved differences between recipients and non-recipients.
- Limitations associated with the use of routinely collected data include under-ascertainment of deaths, imperfect measurement of incomes, and a restricted range of covariates.
- The period of follow-up is limited to ten years, so may be insufficient to observe long-term impacts, including life course effects of improving socio-economic conditions in childhood.

For peer review only

For peer review only - http://bmjopen.bmj.com/site/about/guidelines.xhtml

Introduction

Conditional cash transfer (CCT) programmes have been widely implemented since the 1990s in the Americas, aiming to reduce poverty among groups largely excluded from previous social policies[1]. A World Bank Study using data from 79 countries suggested that such programmes have reduced absolute poverty (people living with <= US$1.90 per capita per day) by 36% and relative poverty (the poorest 20% in each country) by 8%[2]. CCT programs impose additional requirements on recipients, beyond a test of income, most commonly a health and/or education component that targets children and pregnant/breastfeeding women[1]. Therefore, the evidence of the impact of cash transfer programmes on health derives largely (but not exclusively) from studies evaluating the impact on food availability, nutrition, child and maternal health[3-5].

In low- and middle-income countries, cardiovascular diseases (CVD) still lead as the number one cause of death among non-communicable diseases (NCD), accounting for 9.6 million deaths in 2017, but mortality rates have been mainly increasing in middle-income countries[6, 7]. A recent systematic review notes low socioeconomic status, high alcohol intake, obesity, diabetes, hypertension, physical inactivity and smoking are the main modifiable factors associated with early mortality due to CVD [8]. Premature mortality (i.e., death among persons 30 to 69 years of age) is an important indicator included in the Sustainable Developmental Goals (SDG) 3.4 target[9] for monitoring the implementation of effective public policies for disease prevention and control[10].

In Brazil, poverty is largely concentrated among women, Blacks, and individuals living in rural areas[11]. The epidemiological transition in the country occurred heterogeneously across regions, and the overall increase in life expectancy was accompanied by a greater decrease in CVD mortality in regions with better socioeconomic conditions[12, 13]. In 2013, a nationwide survey highlighted a higher prevalence of risk factors for CVD (i.e., smoking, heavy alcohol use, physical inactivity and sedentary lifestyle, as well as the lower consumption of fruits and vegetables) among Brazilians from Non-White ethnic backgrounds and those with lower education, illustrating the relationship between poverty and racial/ethnic, gender and income disparities in health[14].

The Bolsa Familia Program (BFP), the world's largest CCT, benefits over 13 million families a year and has helped to reduce poverty and inequality in Brazil[15-17]. Although the programme has had large effects on child and maternal mortality, its impact on NCDs, especially on cardiovascular deaths, remains unknown. Therefore, the aims of our study are to investigate the effects of the BFP on CVD (ischaemic heart disease and cerebrovascular disease) mortality and all-cause mortality, and to assess how these effects differ by socioeconomic position, race/ethnicity, urbanicity and region.

Our detailed objectives are:

1. To estimate the causal effect of BFP on premature cardiovascular disease mortality

For peer review only - http://bmjopen.bmj.com/site/about/guidelines.xhtml

2. To estimate the causal effect of BFP on premature ischaemic heart disease and cerebrovascular disease mortality

3. To estimate the causal effect of BFP on all-cause premature mortality.

4. To investigate whether the causal effects of BFP on cardiovascular and all-cause mortality differ by population subgroups, including gender, educational attainment, race/ethnicity, geographical region, urbanicity, and socioeconomic position.

5. To explore how combinations of selected social characteristics influence the causal effects of BFP on the above outcomes, adopting an intersectionality perspective.

Methods and analysis

Study design

This study will be analysed as a retrospective, dynamic and open cohort, linking data from the baseline registries of individuals in the 100 Million Brazilian Cohort, with data on Bolsa Familia Program intervention receipt and individual level mortality records for the whole country.

Intervention

We report key implementation characteristics as per the TIDieR-PHP template[18]. BFP was implemented in 2004 and involves cash payments to poor families within Brazil, conditional on educational and health requirements [16]. Under BFP, cash benefits are preferentially paid to women in qualifying households. To be eligible, households must be registered in the Brazil's National Registry for Social Programmes "Cadastro Único" (CadUnico), and have a household income below a defined extreme poverty threshold (monthly per capita household income ≤ BRL77 in 2014 (approximately USD19)) or poverty threshold (monthly per capita household income ≤ BRL154 in 2014 (approximately USD39)). A number of changes to income thresholds for eligibility to BFP have occurred from 2004 onwards (Table 1). Extremely poor families receive a fixed benefit of BRL77 plus additional amounts for pregnant women, children and adolescents. Families defined as poor receive the supplements for pregnant women, children and any adolescents in the household, but not the fixed benefit. In the first years of BFP implementation, indigenous and quilombola communities (old African settlements) were prioritized to start receiving the benefit[16]. Receipt of benefit is conditional on families meeting certain qualifying conditions: pregnant women must access prenatal care; children aged 0-5 years and breastfeeding women must undergo monitoring by health professionals (vaccination and health check-ups at home or in the nearest primary healthcare centre); and school-age children must attend school for at least 85% of school-days. If individuals no longer meet the inclusion criteria, ie., if they improve their socioeconomic status, do not meet the conditionalities or do not update the registry every two years, the benefit will only continue for two more years. Nevertheless, non-compliant

For peer review only - http://bmjopen.bmj.com/site/about/guidelines.xhtml

families are thought to be more vulnerable and, in these cases, receive a visit of a social worker that will help families' compliance and their maintenance in the programme[19].

Logic model

We created a logic model (Figure 1), informed by the existing literature, to describe the hypothesised mechanisms through which BFP may have an effect on CVD outcomes. We identified pathways that are likely to operate through material impacts, education and health conditionalities, and by fostering social inclusion, as well as distinguishing short, medium and long-term timescales. Possible short term impacts include changes in nutritional status in adults, through reduced salt and ultra-processed foods intake, increased consumption of foods with high nutrient content, and increases in overall energy intake [20-25]. BFP can also affect the socioeconomic conditions of the household, including more working hours, better jobs and higher income[26-28]. Effects of CCTs that may be observed at in the medium term include those intermediated by the health conditionalities of BFP, such as increased health care utilization[29-31]. Possible long term impacts include those stimulated by reductions in early life and cumulative socioeconomic adversity[32, 33], improved access to education and upward social mobility[34, 35].

Other potential impacts include the cumulative effects of better nutrition, reduced chronic stress and improved access to primary health services[21, 36-38], which can contribute to the control of hypertension, obesity and diabetes and longer survival following acute CVD events[39-41]. We hypothesize that inclusion of BFP beneficiary families in the Family Health Program might promote early CVD diagnosis and better care[42], even though Brazil has a Universal Healthcare System (SUS) and access to free hypertension drugs has substantially increased over recent decades[43]. Also, by providing an income primarily to women, and being accessible to disadvantaged ethno-racial groups, BFP may also contribute directly to reducing race and gender, as well as socio-economic, inequalities in CVD mortality[44-49].

Datasets

Our sample comprises members of families who applied for BFP or other social benefits from 2001 to 2015 registered in CadUnico, combined as part of the baseline of *The 100 Million Brazilian Cohort*[50]. We deterministically linked the baseline information of individuals from the 100 Million Brazilian Cohort (2001-2015) with BFP data (2004-2015) and used a semi-deterministic linkage based on five identifiers (name, sex, year of birth, name of the mother and municipality of residency) to link with individual level mortality records from the Mortality Information System (SIM) (2001-2015) [50]. From 2001 to 2015, over 114 million individuals applied for social benefits, but the final deidentified dataset will include information on the 92 million individuals that applied between 2004 and 2015 (Figure 2).

Sociodemographic variables

The baseline of the 100 Million Brazilian Cohort includes a range of sociodemographic variables collected at individuals' first application in CadÚnico, and includes household

For peer review only - http://bmjopen.bmj.com/site/about/guidelines.xhtml

income, gender, age, race/ethnicity, geographical region and urban-rural classification, housing characteristics and education (Table 2). As the cohort was built from different versions of CadÚnico (ie., version 6 from 2001-2010 and version 7 from 2011-2015), the baseline contain variables that are common to the two versions and those that are only available in one of them. Also, completeness varies widely between variables (0-10% in the selected variables) (Table 2) and over time. Our cohort also contains baseline household monthly per capita income, which was calculated to reflect BFP eligibility criteria and comprises the sum of all household members' income from work, donations, pension and other benefits in the past month of registration, divided by the number of individuals living in the household[51]. For individuals that applied during or after 2011, the work component from the monthly per capita income was calculated as the lowest value of either the total individual income from work in the past month or the sum of an individual's income from work in the past 12 months divided by 12.

Housing characteristics (such as number of rooms within the dwelling, household building material, presence of running water and sanitation) also provide a direct measure of material circumstances, which is less variable over time than income. Highest educational attainment reflects socioeconomic position in early adulthood, since for most people it remains constant after the age of 25 years. As is the case for most administrative datasets, there have been a number of changes in the data collection processes used by the BFP administrators. Changes in the collected variables were harmonized between the different versions when possible but kept separate when there were substantial differences between the studied years. Harmonization was performed by the Cidacs/Fiocruz team when creating the 100 Million Brazilian Cohort and documentation is available online[52].

The extreme poverty and poverty thresholds for BFP eligibility have changed over time at a similar rate (e.g., from 2004 to 2007, extreme poverty criteria changed from ≤50 to ≤60 and poverty criteria changed from ≤100 to ≤120, corresponding to a 20% increase between 2004 and 2007) (Table 1). To account for these changes over time, we will standardize the monthly per capita household income to the 2014 threshold so that we can use a single cut-off value in the analysis for all years. We will multiply the household per capita income value by 1.54 if individuals apply to BFP prior to 28th December 2007, by 1.283 if individuals apply to BFP between 28th December 2007 and 1st September 2009, and by 1.1 if they apply between 1st September 2009 and 31st May 2014 (See Table 1, column 5).

Bolsa Familia Program

The BFP data will provide information on the date each family member started and finished receiving BFP benefit from 1st January 2004 to 31st December 2015. For each individual we will include information on the first and last date they received BFP benefit.

Mortality Information System

Deaths within Brazil are subject to certification by medical professionals so the causes of death (using ICD-10 codes) can be ascertained reasonably precisely. Despite the significant and continuous improvement of data quality over time, regional disparities

For peer review only - http://bmjopen.bmj.com/site/about/guidelines.xhtml

remain with the worst quality in the poorest regions and those with worse health care[53, 54]. In 2000, 14.3% of all deaths corresponded to ill-defined causes and this proportion varied from 28.4% in the Northeast to 6.3% in the South. Since 2005, the Ministry of Health has initiated several actions aimed at improving the quality of mortality information with an emphasis on the North and Northeast regions. By 2010, the proportion of ill-defined causes had dropped to 8.6%, but regional disparities remain[55].

Data analysis plan

Data cleaning and preparation

The 100 Million Brazilian Cohort was cleaned and the variables standardized according to strict protocols developed by Cidacs/Fiocruz. We will start by checking the linkage quality between the 100 Million Brazilian cohort and mortality, check data quality for obvious errors by inspecting the distribution of variables, will investigate the presence of missing data, and make comparisons across similar variables to identify inconsistencies. We will also explicitly check for inconsistencies in data quality between geographical regions and over time. In the presence of substantial discrepancies between regions in the ascertainment of recording of cause of deaths, we shall stratify the analyses by region. Following initial checking and further data cleaning if necessary, we will develop derived variables harmonised over time and will maintain a detailed record and reproducible syntax for the process.

Definitions of exposures and outcomes

As BFP was first implemented in 2004, we will only include in our analysis individuals who registered to the 100 Million Brazilian cohort baseline on the 1st January 2004 onwards (Figure 2, Table 3). Since many applicants will go on to receive BFP eventually, we will allocate exposure status based on whether they are a recipient within different time intervals following first application (Figure 3). Approaches to classifying exposure status will include BFP recipients being defined as those who start receiving the benefit within a given amount of time after registering in the 100 Million Brazilian Cohort baseline. As BFP receipt may vary over time, we will define exposure status on the basis of first receipt. Once a household starts receiving BFP, individuals within the household will continue to be allocated to the exposed group (irrespective of subsequent changes to BFP status).

To evaluate all-cause and cardiovascular mortality among individuals for all ages, the follow-up time (in years) for each individual will start in different time sets after they first apply to 100 Million Brazilian cohort (i.e., after the definition of individuals according to exposure status) and will end at death by any cause or on 31st December 2015. To evaluate all-cause and cardiovascular premature death, follow-up time (in years) for each individual will start when they first apply to the 100 Million Brazilian cohort or, for individuals below 30 years old, on the date they complete 30 years of age. For this analysis, follow-up time will end at death, end of follow-up (31st December 2015), or on reaching 70 years of age.

Our primary outcome is premature cardiovascular disease mortality among adults (ICD codes I00-I99), defined as deaths occurring between 30 and 69 years of age. We shall also

For peer review only - http://bmjopen.bmj.com/site/about/guidelines.xhtml

estimate models for all-ages and premature all-cause mortality to check the sensitivity of our overall approach to the analysis, and for subgroups of cardiovascular death, including ischaemic heart disease (I20-I25), and cerebrovascular disease (I60-I69) (see Figure 2).

Analysis

The final choice of methods for identifying the impact of BFP on CVD mortality will depend on initial exploratory analyses of the exposure and covariate data available in the 100 Million Brazilian Cohort. We will stratify the analyses by year of application (<2010 and ≥2011) to reflect the changes in the income calculation in both periods, which is the main eligibility criteria for BFP receipt.

For individuals that apply ≥2011, for which income data are higher quality (i.e., preliminary data cleaning showed that >75% of individuals had a monthly per capita income <BRL1/USD0.25 prior to 2011), we can use a regression discontinuity design (RDD). RDD uses a threshold in a continuous ('forcing') variable that determines exposure to an intervention to distinguish exposed and unexposed units. The key identifying assumption is that units within a narrow range of values ('bandwidth') of this forcing variable either side of the threshold value will be similar in characteristics other than exposure, so that differences in outcomes can be interpreted as effects of the intervention[56].

Standardized monthly per capita household income will be used as the forcing variable, and we will conduct the analysis for only for the poverty threshold (≤BRL154 / USD39 in 2014), where we have indicative of discontinuity in the probability of receiving BFP given the monthly per capita income. Because eligibility for BFP depends partially on factors other than income, the probability of receipt will not change from 0 to 1 at the income threshold. We shall apply a fuzzy RDD, using two stage least squares, to model receipt of BFP as a function of income and the threshold in the first stage, and fitting the modelled probabilities of receipt as predictors in the outcome models.

We will estimate the effect of BFP on each of the study outcomes using Poisson or Cox proportional hazards survival models, accounting for the length of follow-up of each individual in the cohort[45, 57]. Since individuals are nested within a household, we will use multilevel models to account for the lack of independence of observations[45]. In addition, to test for differences in the effect of BFP by subgroup, we will stratify the analyses by geographical region (South, Southeast, Central-west, North and Northeast) and gender (male and female), household structure, highest educational attainment, housing conditions, urbanicity and municipality level deprivation measures.

One important problem with validity in studies using RDD is manipulation of assignment, for example by applicants understating their income. We shall plot histograms of income to test for smoothness at the thresholds, and scatterplots of covariates against income to check for any bunching below the thresholds that might indicate manipulation, and apply the McCrary test if there is any visual indication of manipulation[58].

We shall perform a series of robustness checks and sensitivity analyses[59]. We shall use plots of the outcomes against income to decide the most appropriate functional form. Given the large numbers available for analysis in the 100 Million Brazilian Cohort, we

For peer review only - http://bmjopen.bmj.com/site/about/guidelines.xhtml

expect to be able to restrict the analyses to households within a narrow range of income either side of the threshold, and to estimate models within a range of bandwidths to test the robustness of the assumption of local linearity, to potentially increase the generalizability of our findings and to compare with further analysis including propensity score based methods.

To improve the robustness of our results, we will perform additional analyses: i. restricted to a subgroup of individuals whose treatment has not varied over time (i.e., excluding those who stopped receiving BFP treatment); ii. restricting the follow-up time to shorter periods in which socioeconomic conditions are less likely to have varied over time; iii. exploring the possibility of treatment contamination occuring when untreated individuals start receiving the BFP, and iv. removing families with zero income or restricting the analysis to individuals that are more likely to receive the treatment (e.g., monthly per capita income below a certain threshold). To test if the effect of BFP on all-cause mortality is independent of BFP effect on homicide and other external causes of death[60, 61], we shall re-estimate the effect excluding external causes of death.

In addition, we will test the robustness of our local effect estimates adjusting the survival models for age or other strong potential confounders. We shall also repeat the analyses in geographical areas with more and less reliable mortality data, e.g. with different proportions of ill-defined causes of death and underreported mortality.

Aside from RDD, we will estimate the effect of BFP on all-cause and cardiovascular mortality using propensity score-based methods. Propensity score methods seek to reduce confounding through the comparison of units that have similar probabilities to be exposed or unexposed to the intervention, given observed characteristics[62]. We will use risk-set matching to calculate the probability of receiving BFP over time, given the baseline demographic and socioeconomic variables of the 100 Million Cohort[63, 64]. We will match BFP and non-BFP participants overall or within strata using the propensity score. Matching methods will include nearest neighbour matching using adequate calliper and replacement. We will estimate the average treatment effect on the treated (ATT) of BFP on overall mortality and cardiovascular outcomes using Cox or Poisson models. Alternative approaches to deal with changes in BFP status over time will include truncating the follow-up period to a certain number of years, excluding households that do not receive BFP for a minimum number of years and considering the treatment indicator as varying over time.

To verify the hypothesis that matching could be pruning similar individuals, we will estimate in the unmatched cohort the ATT and the average treatment effect (ATE) using survival models weighted by the inverse of the probability of receiving the treatment (inverse probability of the treatment weighting - IPTW). Also, we will estimate both ATT and ATE in quintiles or deciles of propensity score strata. For all the methods that rely on observable covariates, we will check balance of covariates between the intervention and control groups.

To deal with missing data, we will start by exploring the missingness pattern of covariates over time in our study population. Given the size of our sample and the complexity of

For peer review only - http://bmjopen.bmj.com/site/about/guidelines.xhtml

causal inference methods, we are unable to implement multiple imputation. For the development of the propensity score, we will try to limit inclusion of covariates to those with a relatively low percentage of missing values (e.g., <5%). For variables which have higher levels of missingness but which are strongly informative of intervention receipt, we will include a missing indicator for that variable. In addition, we will perform a sensitivity analysis using only individuals without missing data in the covariates of interest (i.e., complete case analysis).

Our preference, as informed by the MRC's guidance on the evaluation of natural experiments[45], is to use both RDD and propensity score based methods, since each method relies on different assumptions (e.g., no unobserved confounding for propensity score based methods and as-if random allocation at the eligibility cut-off for BFP in the case of RDD). Obtaining consistent results from the two approaches for individuals that applied ≥2011 will, along with the other robustness checks, strengthen confidence in our inferences about impact. For each of the analyses we will evaluate whether the assumptions of the corresponding methodology are tenable. The exploratory analyses and decisions leading to the final specification of the analyses will be fully documented and reported alongside the findings.

Effect modification and intersectionality

We will investigate differential impacts of BFP on mortality by including interaction terms within the models and by stratifying the analyses to provide estimates for population subgroups defined by gender, race/ethnicity, urbanicity and socioeconomic position (education and area-level deprivation). To investigate intersectionality, we will test for effect measure modification on both an additive scale and a multiplicative scale [65, 66]. We are especially interested in exploring variation according to combinations of characteristics, such as gender and race/ethnicity (Figure 4)[65]. Therefore, we will create a single categorical variable that incorporates the two concepts of interest and estimate the relative excess risk due to interaction[66]. To study intersectionality on a multiplicative scale, we will fit interaction terms between variables representing the two concepts. For further subgroup analyses, we shall submit a full specification of the analysis of interest to the SPHI project steering group before data analysis begins and report the results of all analyses rather than selecting according to size or significance of effects.

Time schedule

The time schedule for the analysis is described in Table 4.

Patient and Public Involvement

Patients and the public were not involved in this study.

Ethics and dissemination

The 100 Million Brazilian Cohort study was approved by the ethics committee of Gonçalo Muniz Institute - Oswaldo Cruz Foundation (1.612.302) and the specific aims of this

For peer review only - http://bmjopen.bmj.com/site/about/guidelines.xhtml

project were submitted for ethical approval to the same ethics committee. The University of Glasgow Medical, Veterinary & Life Sciences College Ethics Committee also approved the study (project number: 200190001). All data are linked in a safe room with access to restricted people only. After data are linked and linkage accuracy is calculated, researchers will have full access to the deidentified dataset. The dataset will be accessed by researchers upon application to a data curation committee with a detailed analysis plan. The dataset will receive a Digital Object Identifier System (DOI), and full specification of how the dataset was created will be available online. All manuscripts will be published in open access journals. The study is part of a larger project, the NIHR Global Health Research Group on Social Policy and Health Inequalities, which supports a communications group responsible for producing summaries of the published research results for managers, policymakers and the broader public.

Author contributions: JP, PC, EA and SVK wrote the first draft of the protocol. MA, LA, SA, LS, MLB and AL contributed with additional material. All authors participated with the discussions during the development of this protocol and reviewed the final version.

Funding statement: This research was funded by the National Institute for Health Research (NIHR) (GHRG /16/137/99) using UK aid from the UK Government to support global health research. The views expressed in this publication are those of the author(s) and not necessarily those of the NIHR or the UK Department of Health and Social Care. The Social and Public Health Sciences Unit is core funded by the Medical Research Council (MC_UU_12017/13) and the Scottish Government Chief Scientist Office (SPHSU13). SVK is funded by a NHS Research Scotland Senior Clinical Fellowship (SCAF/15/02). Cidacs/Fiocruz is supported by grants from CNPq/MS/Gates Foundation (401739/2015-5) and the Wellcome Trust, UK (202912/Z/16/Z).

Competing interests statement: The authors declare no competing interests.

References

1. Bastagli F. Poverty, inequality and public cash transfers: lessons from Latin America. 2010.
2. Ivaschenko O, Alas CPR, Novikova M, Romero C, Bowen T, Chu LJ. The State of Social Safety Nets 2018. Washington: The World Bank; 2018.
3. Bastagli F, Hagen-Zanker J, Harman L, Barca V, Sturge G, Schmidt T. The Impact of Cash Transfers: A Review of the Evidence from Low- and Middle-income Countries. Journal of Social Policy. 2019;48(3):569-94. doi: 10.1017/s0047279418000715.
4. Fiszbein A, Schady NR. Conditional cash transfers: reducing present and future poverty: The World Bank; 2009.
5. Gloria M, Laura B. Evaluating the impact of conditional cash transfer programs: lessons from Latin America. Policy Research Working Paper Series. 2003.
6. PAHO. World Heart Day: Cardiovascular Diseases Cause 1.9 Million Deaths a Year in the Americas: Pan American Health Organization (PAHO); 2019 [cited 2019 06/08//2019]. Available from: https://www.paho.org/.

For peer review only - http://bmjopen.bmj.com/site/about/guidelines.xhtml

7. GBD Compare [Internet]. IHME, University of Washington. 2019 [cited 24/09/2019]. Available from: http://vizhub.healthdata.org/gbd-compare.
8. Stringhini S, Carmeli C, Jokela M, Avendaño M, Muennig P, Guida F, et al. Socioeconomic status and the 25× 25 risk factors as determinants of premature mortality: a multicohort study and meta-analysis of 1· 7 million men and women. The Lancet. 2017;389(10075):1229-37.
9. Nations U. Transforming our world: The 2030 agenda for sustainable development. New York: United Nations, Department of Economic and Social Affairs. 2015.
10. Ordunez P, Prieto-Lara E, Pinheiro Gawryszewski V, Hennis AJM, Cooper RS. Premature Mortality from Cardiovascular Disease in the Americas – Will the Goal of a Decline of "25% by 2025" be Met? PLOS ONE. 2015;10(10):e0141685. doi: 10.1371/journal.pone.0141685.
11. Paiva L. Poverty and Inequality Reduction in Brazil–A Parenthesis in History or the Road Ahead? Iberoamericana–Nordic Journal of Latin American and Caribbean Studies. 2016;45(1).
12. Marinho F, de Azeredo Passos VM, Malta DC, França EB, Abreu DM, Araújo VE, et al. Burden of disease in Brazil, 1990–2016: a systematic subnational analysis for the Global Burden of Disease Study 2016. The Lancet. 2018;392(10149):760-75.
13. Brant LCC, Nascimento BR, Passos VMA, Duncan BB, Bensenõr IJM, Malta DC, et al. Variações e diferenciais da mortalidade por doença cardiovascular no Brasil e em seus estados, em 1990 e 2015: estimativas do Estudo Carga Global de Doença. Revista Brasileira de Epidemiologia. 2017;20:116-28.
14. Barros MBdA, Lima MG, Medina LdPB, Szwarcwald CL, Malta DC. Social inequalities in health behaviors among Brazilian adults: National Health Survey, 2013. International journal for equity in health. 2016;15(1):148-. doi: 10.1186/s12939-016-0439-0. PubMed PMID: 27852275.
15. Soares S, De Souza PHGF, Osório RG, Silveira FG. Os impactos do benefício do Programa Bolsa Família sobre a desigualdade e a pobreza. In: IPEA, editor. Bolsa Família 2003-2010: avanços e desafios. 2010. Brasilia2010. p. 27-52.
16. Campello T, Neri MC. Programa Bolsa Família: uma década de inclusão e cidadania. Brasília: Ipea. 2013.
17. Brasil, MDS. Relatórios de Informações Sociais - Bolsa Família e Cadastro Único: Ministerio da Cidadania (MDS); 2020 [cited 2020 31/03/2020]. Available from: https://aplicacoes.mds.gov.br/sagi/RIv3/geral/index.php?relatorio=153&file=entrada#.
18. Campbell M, Katikireddi SV, Hoffmann T, Armstrong R, Waters E, Craig P. TIDieR-PHP: a reporting guideline for population health and policy interventions. bmj. 2018;361:k1079.
19. Soares FV. Brazil's Bolsa Família: A Review. Economic and Political Weekly. 2011;46(21):55-60.
20. Coelho PL, Melo A. The impact of the "Bolsa Familia" Program on household diet quality, Pernambuco State, Brazil. Cienc Saude Coletiva. 2017;22(2):393-402. doi: 10.1590/1413-81232017222.13622015. PubMed PMID: WOS:000394147100007.
21. Martins APB, Monteiro CA. Impact of the Bolsa Família program on food availability of low-income Brazilian families: A quasi experimental study. BMC public health. 2016;16(1). doi: 10.1186/s12889-016-3486-y.
22. Ferrario MN. The impacts on family consumption of the Bolsa Familia subsidy programme. Cepal Rev. 2014;(112):147-63. PubMed PMID: WOS:000340076200008.
23. Sperandio N, Rodrigues CT, Franceschini SdCC, Priore SE. Impacto do Programa Bolsa Família no consumo de alimentos: estudo comparativo das regiões Sudeste e Nordeste do Brasil. Ciênc Saúde Colet. 2017;22(6):1771-80.
24. Sperandio N, Rodrigues CT, Franceschini SDC, Priore SE. Impact of the Bolsa Familia Program on energy, macronutrient, and micronutrient intakes: Study of the Northeast and Southeast. Rev Nutr. 2016;29(6):833-44. doi: 10.1590/1678-98652016000600008. PubMed PMID: WOS:000391443800009.

For peer review only - http://bmjopen.bmj.com/site/about/guidelines.xhtml

25. Baptistella JCF. Avaliação de programas sociais: uma análise do impacto do Bolsa Família sobre o consumo de alimentos. 2012.
26. Cabral CS, Lopes AG, Lopes JM, Vianna RPT. Food security, income, and the Bolsa Família program: A cohort study of municipalities in Paraíba State, Brazil, 2005-2011. Cad Saude Publica. 2014;30(2):393-402. doi: 10.1590/0102-311X00140112.
27. Barbosa ALNH, Corseuil CHL. Conditional cash transfer and informality in Brazil. IZA J Labor Dev. 2014;3(1). doi: 10.1186/s40175-014-0024-0.
28. Nazareno LdA. O programa bolsa família e o mercado de trabalho informal: Uma análise de impacto da ação Brasil carinhoso. Econ Apl. 2016;20(4):457-71. doi: 10.11606/1413-8050/ea153891.
29. Fernald LC, Gertler PJ, Hou X. Cash component of conditional cash transfer program is associated with higher body mass index and blood pressure in adults. The Journal of nutrition. 2008;138(11):2250-7.
30. Gertler P. The impact of PROGRESA on health. 2000 2000. Report No.
31. Witvorapong N, Foshanji AI. The impact of a conditional cash transfer program on the utilization of non-targeted services: Evidence from Afghanistan. Social Science & Medicine. 2016;152:87-95.
32. Pollitt RA, Rose KM, Kaufman JS. Evaluating the evidence for models of life course socioeconomic factors and cardiovascular outcomes: a systematic review. BMC public health. 2005;5(1):7.
33. Hardy R, Lawlor DA, Kuh D. A life course approach to cardiovascular aging. Future cardiology. 2015;11(1):101-13.
34. de Brauw A, Gilligan DO, Hoddinott J, Roy S. The Impact of Bolsa Familia on Schooling. World Development. 2015;70:303-16. doi: 10.1016/j.worlddev.2015.02.001. PubMed PMID: WOS:000351969500023.
35. Glewwe P, Kassouf AL. The impact of the Bolsa Escola/Familia conditional cash transfer program on enrollment, dropout rates and grade promotion in Brazil. J Dev Econ. 2012;97(2):505-17. doi: 10.1016/j.jdeveco.2011.05.008.
36. Simões AA, Sabates R. The Contribution of Bolsa Família to the educational achievement of economically disadvantaged children in Brazil. Int J Educ Dev. 2014;39:141-56.
37. Santos TMd, Silva SSdC, Koller SH. Evaluation of Amazon Riverine Beneficiaries about Bolsa Família Program. Psicologia: Teoria e Pesquisa. 2017;33.
38. Shei A, Costa F, Reis MG, Ko AI. The impact of Brazil's Bolsa Família conditional cash transfer program on children's health care utilization and health outcomes. BMC Int Health Hum Rights. 2014;14(1). doi: 10.1186/1472-698X-14-10.
39. Chandola T, Britton A, Brunner E, Hemingway H, Malik M, Kumari M, et al. Work stress and coronary heart disease: what are the mechanisms? European heart journal. 2008;29(5):640-8. Epub 2008/01/25. doi: 10.1093/eurheartj/ehm584. PubMed PMID: 18216031.
40. Chandola T, Brunner E, Marmot M. Chronic stress at work and the metabolic syndrome: prospective study. Bmj. 2006;332(7540):521-5. Epub 2006/01/24. doi: 10.1136/bmj.38693.435301.80. PubMed PMID: 16428252; PubMed Central PMCID: PMCPMC1388129.
41. Hjelm L, Handa S, de Hoop J, Palermo T. Poverty and perceived stress: Evidence from two unconditional cash transfer programs in Zambia. Social science & medicine (1982). 2017;177:110-7. Epub 2017/02/09. doi: 10.1016/j.socscimed.2017.01.023. PubMed PMID: 28167339.
42. Rasella D, Harhay MO, Pamponet ML, Aquino R, Barreto ML. Impact of primary health care on mortality from heart and cerebrovascular diseases in Brazil: a nationwide analysis of longitudinal data. Bmj. 2014;349:g4014. Epub 2014/07/06. doi: 10.1136/bmj.g4014. PubMed PMID: 24994807; PubMed Central PMCID: PMCPMC4080829.

For peer review only - http://bmjopen.bmj.com/site/about/guidelines.xhtml

43. Emmerick ICM, do Nascimento JM, Pereira MA, Luiza VL, Ross-Degnan D. Farmácia Popular Program: changes in geographic accessibility of medicines during ten years of a medicine subsidy policy in Brazil. Journal of pharmaceutical policy and practice. 2015;8(1):10.
44. Attanasio O, Battistin E, Mesnard A. Food and Cash Transfers: Evidence from Colombia. The Economic Journal. 2012;122(559):92-124. doi: 10.1111/j.1468-0297.2011.02473.x.
45. Tommasi D. Control of resources, bargaining power and the demand of food: Evidence from PROGRESA. Journal of Economic Behavior & Organization. 2019;161:265-86.
46. Brauw A, Gilligan DO, Hoddinott J, Roy S. The Impact of Bolsa Famı´lia on Women's Decision-Making Power. World Development. 2014;59:487-504. doi: 10.1016/j.worlddev.2013.02.003.
47. Carloto C, Mariano SA. Empoderamento, trabalho e cuidados: mulheres no programa bolsa família. Textos & Contextos (Porto Alegre). 2012;11(2).
48. Bell CN, Thorpe RJ, Jr., Bowie JV, LaVeist TA. Race disparities in cardiovascular disease risk factors within socioeconomic status strata. Annals of epidemiology. 2018;28(3):147-52. Epub 2018/01/11. doi: 10.1016/j.annepidem.2017.12.007. PubMed PMID: 29317176.
49. Brondolo E. Considering social processes when examining mediators of the effects of discrimination on health. Social Science & Medicine. 2018;215:160-2. doi: https://doi.org/10.1016/j.socscimed.2018.08.004.
50. Ali MS, Ichihara MY, Lopes LC, Barbosa GCG, Pita R, Carreiro RP, et al. Administrative Data Linkage in Brazil: Potentials for Health Technology Assessment. Front Pharmacol. 2019;10:984-. doi: 10.3389/fphar.2019.00984. PubMed PMID: 31607900.
51. Brasil. Perguntas Frequentes sobre Dados do Cadastro Único para Programas Sociais. In: (Senarc) MdDSMSNdRdC, editor. Brasilia2011.
52. Barreto ML, Ichihara MYT, Almeida BdA, Barreto ME, Cabral L, Fiaccone RL, et al. The Centre for Data and Knowledge Integration for Health (CIDACS): Linking Health and Social Data in Brazil. 2019.
53. Lima EECd, Queiroz BL. Evolution of the deaths registry system in Brazil: associations with changes in the mortality profile, under-registration of death counts, and ill-defined causes of death. Cadernos de Saúde Pública. 2014;30:1721-30.
54. Junior DFM. The Importance of Reduction in the Registry of Deaths to Ill-Defined Causes and their Impact on Mortality Profile: The Brazilian Experience and its Implications to International Health Security. Contemporary Developments and Perspectives in International Health Security-Volume 1: IntechOpen; 2020.
55. França E, Teixeira R, Ishitani L, Duncan BB, Cortez-Escalante JJ, Morais Neto OLd, et al. Ill-defined causes of death in Brazil: a redistribution method based on the investigation of such causes. Rev Saude Publica. 2014;48:671-81.
56. Bor J, Moscoe E, Mutevedzi P, Newell M-L, Bärnighausen T. Regression discontinuity designs in epidemiology: causal inference without randomized trials. Epidemiology (Cambridge, Mass). 2014;25(5):729.
57. Tchetgen EJT, Walter S, Vansteelandt S, Martinussen T, Glymour M. Instrumental variable estimation in a survival context. Epidemiology (Cambridge, Mass). 2015;26(3):402.
58. Imbens GW, Lemieux T. Regression discontinuity designs: A guide to practice. Journal of econometrics. 2008;142(2):615-35.
59. Lee DS, Lemieux T. Regression discontinuity designs in economics. Journal of economic literature. 2010;48(2):281-355.
60. Alvarez J-A, Aburto JM, Canudas-Romo V. Latin American convergence and divergence towards the mortality profiles of developed countries. Population Studies. 2020;74(1):75-92.
61. Machado DB, Rodrigues LC, Rasella D, Lima Barreto M, Araya R. Conditional cash transfer programme: Impact on homicide rates and hospitalisations from violence in Brazil. PloS one. 2018;13(12):e0208925.
62. Rubin DB. Estimating causal effects from large data sets using propensity scores. Annals of internal medicine. 1997;127(8_Part_2):757-63.

For peer review only - http://bmjopen.bmj.com/site/about/guidelines.xhtml

63. Watson D, Spaulding AB, Dreyfus J. Risk-set matching to assess the impact of hospital-acquired bloodstream infections. American journal of epidemiology. 2019;188(2):461-6.
64. Danaei G, Rodríguez LAG, Cantero OF, Logan RW, Hernán MA. Electronic medical records can be used to emulate target trials of sustained treatment strategies. Journal of clinical epidemiology. 2018;96:12-22.
65. Bauer GR. Incorporating intersectionality theory into population health research methodology: challenges and the potential to advance health equity. Social science & medicine. 2014;110:10-7.
66. Knol MJ, VanderWeele TJ. Recommendations for presenting analyses of effect modification and interaction. International journal of epidemiology. 2012;41(2):514-20.

For peer review only

For peer review only - http://bmjopen.bmj.com/site/about/guidelines.xhtml

TABLES AND FIGURES

Table 1. Eligibility criteria for Bolsa Familia Program (BFP) receipt in Brazil between 2004 and 2015 and income standardization rates.

**Year**	**Extremely poverty eligibility criteria[1]**	**Poverty eligibility criteria[1]**	**Date of change**	**Income standardization rate[2] from 2004 to 2014 thresholds**
2004	≤BRL 50 (USD12.5)	≤BRL 100 BRL (USD26)		Income x 1.540
2007	≤BRL 60 (USD15)	≤BRL 120 BRL (USD30)	28th Dec 2007	Income x 1.283
2009	≤BRL 70 (USD17.5)	≤BRL 140 BRL (USD35)	1st Sept 2009	Income x 1.100
2014	≤BRL 77 (USD19.3)	≤BRL 154 BRL (USD38.5)	1st Jun 2014	Income

[1]Values in USD were calculated based on the following exchange rate: 4BRL=1USD.

[2]For each year (2004, 2007 and 2009), the standardized rate was estimated by dividing the poverty (or extreme poverty) criteria from 2014 by the poverty criteria (or extreme poverty) from that year.

Table 2. Key variables available for the analysis from the 100 Million Brazilian Cohort baseline, from Bolsa Familia dataset and from mortality data.

100 Million Brazilian Cohort baseline	
Individual level variables	
**Age (at application)**	Continuous
**Sex**	Female, Male
**Relationship with the responsible person for the household**	Responsible him/herself, wife/husband, son/daughter, stepson/stepdaughter, grandchildren, parent-in-law, brother/sister, son/daughter-in-law, other relative, not relative.
**Race/Ethnicity**	White, Brown, Black, Asian, Indigenous
**Literacy**	Literate or illiterate.
**Level of education**	Never went to school, Pre-school, literacy school, primary education (first stage - 5 years), primary education (second stage - 4 years), High School, Higher education
**Monthly per capita income[1]**	Sum of income from work[2], donations, pension and others per divided by the number of individuals in the household in the given year.
Family level variables	
**Municipality of family home**	Single identifier for every municipality
**Region of family home**	South, South-east, North, North-east, Central-West
**Location of family home**	Urban, rural
**Housing material**	Brick or cement, Taipa, Wood, Other
**Household type**	Private, improvised but private, collective, others.
**Household water supply**	Public network, Well or natural source, cistern or others
**Sewage disposal system**	Public network, Septic tank, rudimentary tank, ditch, others.

For peer review only - http://bmjopen.bmj.com/site/about/guidelines.xhtml

**Electricity**	Home meter, Community meter, irregular electricity, gas lighting, candlelight, other
**Waste collection**	Public collection system, burned, buried, outdoor disposal, other
**Number of individuals in the household**	Continuous
**Number of rooms in the household (including bathrooms, living room and kitchen if separated by walls)**	Continuous
Bolsa Familia Program variables	
**Benefit starting date**	Date
**Benefit ending date**	Date
**Duration of the benefit receipt**	Time in years
Mortality system information (SIM)	
**Date of death**	Date
**Place of death**	Hospital, other health establishments, household, street, others
**Municipality of death**	Single identifier for every municipality
**Medical assistance**	Yes or No
**Necropsy investigation**	Yes or No
**Main cause of death**	ICD-10 categories

For peer review only - http://bmjopen.bmj.com/site/about/guidelines.xhtml

Table 3. All cause and cardiovascular mortality rates among individuals from the 100 Million Brazilian Cohort (N=92 Million).

	Deaths (x10^3)	Mean yearly mortality rates per 100,000 person-years at risk during the study period		
		**Overall**	**Male**	**Female**
**Overall population (N=92 millions)**				
All cause mortality rate	1,810	286	370	199
Cardiovascular mortality				
All cardiovascular mortality rate (I00-99)	432	68	81	56
Cerebrovascular mortality rate (I60-69)	113	18	20	15
Ischaemic heart diseases mortality rate (I20-25)	117	19	24	13
**Individuals aged 30-69 y (N=34 millions)**				
All cause premature mortality rate	943	482	604	344
Premature cardiovascular mortality				
CVD premature mortality rate (I00-99)	243	124	147	98
Cerebrovascular premature mortality rate (I60-69)	63	32	36	28
Ischaemic heart diseases premature mortality rate (I20-25)	76	39	50	26

For peer review only

Table 4. *Time schedule for evaluation the effect of BFP on all cause and cardiovascular diseases mortality in The 100 Million Brazilian Cohort.*

	2020												2021				
	J	F	M	A	M	J	J	A	S	O	N	D	J	F	M	A	M
Elaboration of the study protocol - submitted 22/4/20	X	X	X	X													
Descriptive data analysis				X	X	X	X	X	X								
Impact of Bolsa Família on all cause and cardiovascular mortality (data analyis)							X	X	X	X	X						
Impact of Bolsa Família on all cause and cardiovascular mortality (writing)											X	X					
Impact of Bolsa Família on all cause and cardiovascular mortality with focus on gender, race and social inequality (data analyis)												X	X	X	X		
Impact of Bolsa Família on all cause and cardiovascular mortality with focus on gender, race and social inequality (writing)															X	X	X
Impact of Bolsa Família all cause and cardiovascular mortality with focus on deprivation index/inequalities (data analyis)												X	X	X	X		
Impact of Bolsa Família all cause and cardiovascular mortality with focus on deprivation index/inequalities (writing)															X	X	X

For peer review only

Fig 1. Logic Model of the potential effect of Bolsa Familia Program (BFP) on all-cause and cardiovascular mortality.

Fig 2. Flowchart of data selection.

Fig 3. Approximate cumulative number and proportion of individuals of all ages receiving BFP over time after start of follow-up for those that apply between 2004-2010 (Cohort 1) (A) and for those that apply between 2011-2015 (Cohort 2) (B).

Fig 4. Main strata to investigate intersectionality regarding gender and race/ethnicity among individuals from all ages from the 100 Million Brazilian Cohort (2004-2015).

For peer review only - http://bmjopen.bmj.com/site/about/guidelines.xhtml

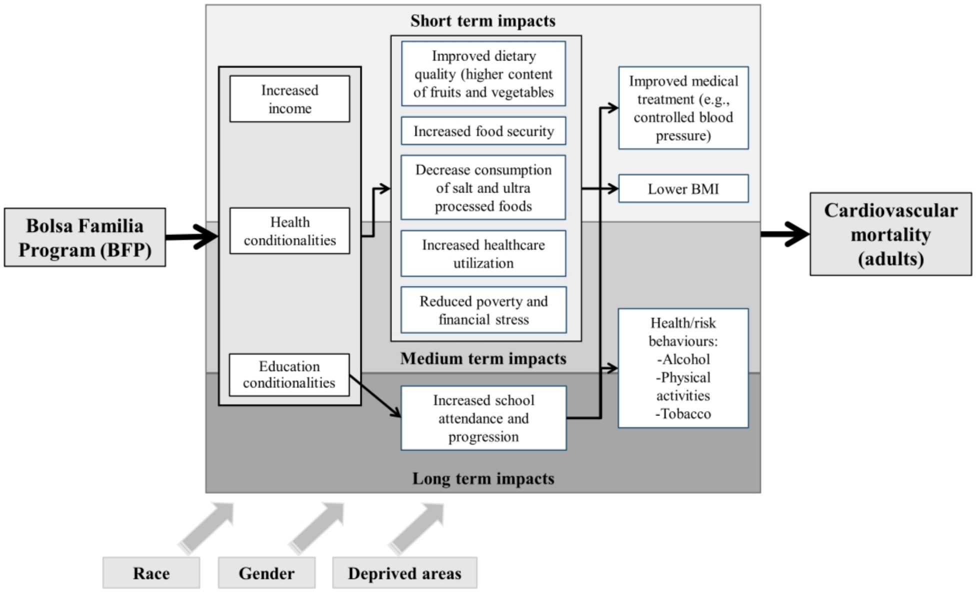

Fig 1. Logic Model of the potential effect of Bolsa Familia Program (BFP) on all-cause and cardiovascular mortality.

279x176mm (300 x 300 DPI)

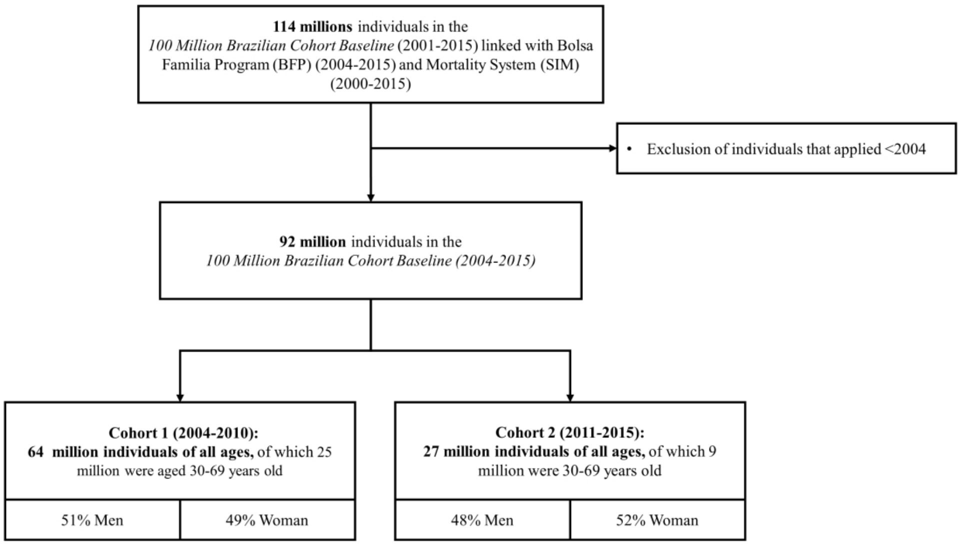

Fig 2. Flowchart of data selection.

270x170mm (300 x 300 DPI)

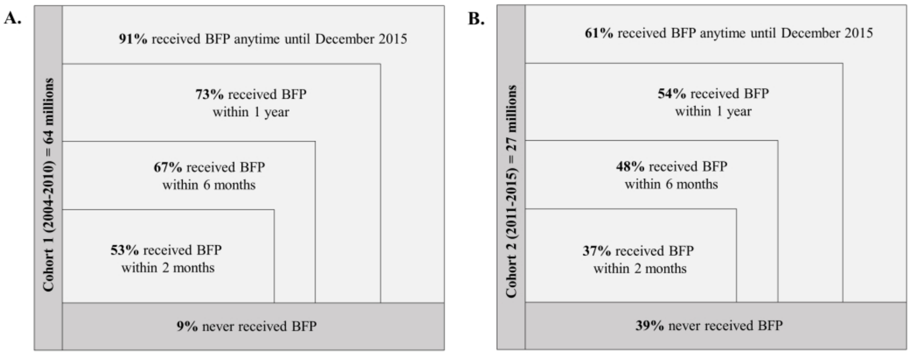

Fig 3. Approximate cumulative number and proportion of individuals of all ages receiving BFP over time after start of follow-up for those that apply between 2004-2010 (Cohort 1) (A) and for those that apply between 2011-2015 (Cohort 2) (B).

338x190mm (300 x 300 DPI)

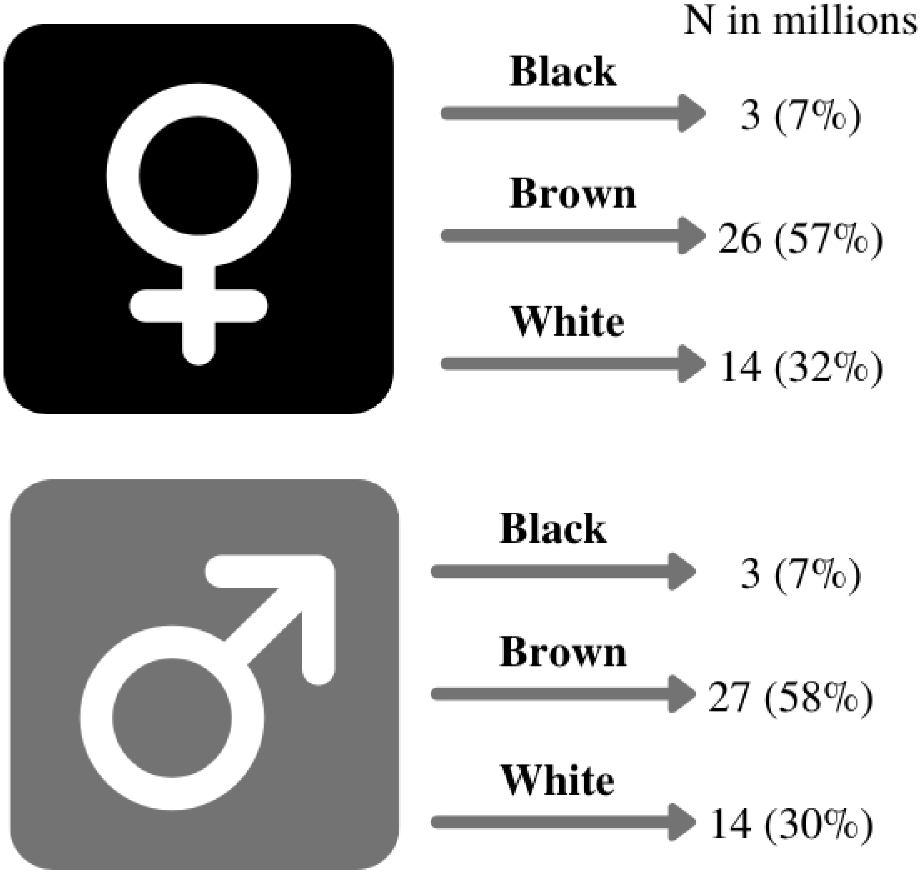

Fig 4. Main strata to investigate intersectionality regarding gender and race/ethnicity among individuals from all ages from the 100 Million Brazilian Cohort (2004-2015).

STROBE Statement—checklist of items that should be included in reports of observational studies

	Item No.	Recommendation	Page No.	Relevant text from manuscript
**Title and abstract**	1	(*a*) Indicate the study's design with a commonly used term in the title or the abstract	1;2	
		(*b*) Provide in the abstract an informative and balanced summary of what was done and what was found	2	
**Introduction**				
Background/rationale	2	Explain the scientific background and rationale for the investigation being reported	4	
Objectives	3	State specific objectives, including any prespecified hypotheses	4-6	
**Methods**				
Study design	4	Present key elements of study design early in the paper	5	
Setting	5	Describe the setting, locations, and relevant dates, including periods of recruitment, exposure, follow-up, and data collection	6	
Participants	6	(*a*) *Cohort study*—Give the eligibility criteria, and the sources and methods of selection of participants. Describe methods of follow-up<br>*Case-control study*—Give the eligibility criteria, and the sources and methods of case ascertainment and control selection. Give the rationale for the choice of cases and controls<br>*Cross-sectional study*—Give the eligibility criteria, and the sources and methods of selection of participants		
		(*b*) *Cohort study*—For matched studies, give matching criteria and number of exposed and unexposed<br>*Case-control study*—For matched studies, give matching criteria and the number of controls per case	6-10	
Variables	7	Clearly define all outcomes, exposures, predictors, potential confounders, and effect modifiers. Give diagnostic criteria, if applicable	8-10	
Data sources/ measurement	8*	For each variable of interest, give sources of data and details of methods of assessment (measurement). Describe comparability of assessment methods if there is more than one group	6-7	
Bias	9	Describe any efforts to address potential sources of bias	9-11	
Study size	10	Explain how the study size was arrived at	6	

Continued on next page

For peer review only - http://bmjopen.bmj.com/site/about/guidelines.xhtml

For peer review only

Quantitative variables	11	Explain how quantitative variables were handled in the analyses. If applicable, describe which groupings were chosen and why	NA
Statistical methods	12	(*a*) Describe all statistical methods, including those used to control for confounding	8-11
		(*b*) Describe any methods used to examine subgroups and interactions	9-11
		(*c*) Explain how missing data were addressed	10
		(*d*) *Cohort study*—If applicable, explain how loss to follow-up was addressed<br>*Case-control study*—If applicable, explain how matching of cases and controls was addressed<br>*Cross-sectional study*—If applicable, describe analytical methods taking account of sampling strategy	
		(*e*) Describe any sensitivity analyses	9-11
**Results**			
Participants	13*	(a) Report numbers of individuals at each stage of study—eg numbers potentially eligible, examined for eligibility, confirmed eligible, included in the study, completing follow-up, and analysed	NA
		(b) Give reasons for non-participation at each stage	NA
		(c) Consider use of a flow diagram	NA
Descriptive data	14*	(a) Give characteristics of study participants (eg demographic, clinical, social) and information on exposures and potential confounders	NA
		(b) Indicate number of participants with missing data for each variable of interest	NA
		(c) *Cohort study*—Summarise follow-up time (eg, average and total amount)	NA
Outcome data	15*	*Cohort study*—Report numbers of outcome events or summary measures over time	NA
		Case-control study—Report numbers in each exposure category, or summary measures of exposure	NA
		Cross-sectional study—Report numbers of outcome events or summary measures	NA
Main results	16	(*a*) Give unadjusted estimates and, if applicable, confounder-adjusted estimates and their precision (eg, 95% confidence interval). Make clear which confounders were adjusted for and why they were included	NA
		(*b*) Report category boundaries when continuous variables were categorized	NA
		(*c*) If relevant, consider translating estimates of relative risk into absolute risk for a meaningful time period	NA

Continued on next page

For peer review only - http://bmjopen.bmj.com/site/about/guidelines.xhtml

Other analyses	17	Report other analyses done—eg analyses of subgroups and interactions, and sensitivity analyses	NA
**Discussion**			
Key results	18	Summarise key results with reference to study objectives	NA
Limitations	19	Discuss limitations of the study, taking into account sources of potential bias or imprecision. Discuss both direction and magnitude of any potential bias	3
Interpretation	20	Give a cautious overall interpretation of results considering objectives, limitations, multiplicity of analyses, results from similar studies, and other relevant evidence	NA
Generalisability	21	Discuss the generalisability (external validity) of the study results	NA
**Other information**			
Funding	22	Give the source of funding and the role of the funders for the present study and, if applicable, for the original study on which the present article is based	11

*Give information separately for cases and controls in case-control studies and, if applicable, for exposed and unexposed groups in cohort and cross-sectional studies.

Note: An Explanation and Elaboration article discusses each checklist item and gives methodological background and published examples of transparent reporting. The STROBE checklist is best used in conjunction with this article (freely available on the Web sites of PLoS Medicine at http://www.plosmedicine.org/, Annals of Internal Medicine at http://www.annals.org/, and Epidemiology at http://www.epidem.com/). Information on the STROBE Initiative is available at www.strobe-statement.org.

For peer review only
